# Genome-wide association studies provide insights into the genetic determination of fruit traits of pear

**DOI:** 10.1038/s41467-021-21378-y

**Published:** 2021-02-18

**Authors:** Ming-Yue Zhang, Cheng Xue, Hongju Hu, Jiaming Li, Yongsong Xue, Runze Wang, Jing Fan, Cheng Zou, Shutian Tao, Mengfan Qin, Bing Bai, Xiaolong Li, Chao Gu, Shan Wu, Xu Chen, Guangyan Yang, Yueyuan Liu, Manyi Sun, Zhangjun Fei, Shaoling Zhang, Jun Wu

**Affiliations:** 1 Centre of Pear Engineering Technology Research, State Key Laboratory of Crop Genetics and Germplasm Enhancement, Nanjing Agricultural University, Nanjing, Jiangsu China; 2 Haixia Institute of Science and Technology, Fujian Agriculture and Forestry University, Fuzhou, China; 3 Wuchang Sand Pear Germplasm, Hubei Academy of Agricultural Sciences, Wuhan, Hubei China; 4 Institute of Crop Sciences, Chinese Academy of Agricultural Sciences, Beijing, China; 5 Boyce Thompson Institute, Cornell University, Ithaca, NY USA; 6 USDA-ARS, Robert W. Holley Center for Agriculture and Health, Ithaca, NY USA

**Keywords:** Agricultural genetics, Genome-wide association studies, Plant breeding

## Abstract

Pear is a major fruit tree crop distributed worldwide, yet its breeding is a very time-consuming process. To facilitate molecular breeding and gene identification, here we have performed genome-wide association studies (GWAS) on eleven fruit traits. We identify 37 loci associated with eight fruit quality traits and five loci associated with three fruit phenological traits. Scans for selective sweeps indicate that traits including fruit stone cell content, organic acid and sugar contents might have been under continuous selection during breeding improvement. One candidate gene, *PbrSTONE*, identified in GWAS, has been functionally verified to be involved in the regulation of stone cell formation, one of the most important fruit quality traits in pear. Our study provides insights into the complex fruit related biology and identifies genes controlling important traits in pear through GWAS, which extends the genetic resources and basis for facilitating molecular breeding in perennial trees.

## Abstract (web summary)

Studies of fruit quality traits in pears are lagging behind the other major fruit trees. Here, the authors conduct GWAS of fruit quality and phenological traits in a panel of 312 sand pear accessions using SNPs called from resequencing data, and reveal the involvement of a lignin formation-related protein in regulating stone cell development.

## Introduction

Pear has been cultivated by human for at least 3000 years^1^. However, traditional breeding procedure of pear normally takes at least 13–15 years because of its long juvenile stage. Molecular breeding is an efficient way to help improve cultivars using pre-selection from large numbers of diverse individuals to identify and introduce precise genetic regions that can be expected to confer desired phenotypic benefits. For example, previous work identified 31 quantitative trait loci (QTLs) for 11 traits based on a pear linkage map constructed with 3241 markers^2^. Amplified fragment length polymorphism (AFLP) markers were identified to be linked to a scab resistance gene in an inter-specific hybrid pear^3^, and fire-blight resistance-related QTLs were identified in European pears^4,5^. Pear harvest time and skin color-related QTLs were also investigated in Japanese pears^6^. However, pear research and breeding have been limited by factors such as the time-consuming process of constructing cross populations for genetic mapping, and restricted genetic diversity.

With the completion of pear whole-genome sequencing, analyzing large natural populations through high-throughput sequencing and genome-wide association studies (GWAS) provides tremendous opportunities for exploring variation alleles and key genes underlying important traits, which can help to develop molecular breeding markers and reveal genetic mechanisms of these traits. In plants, GWAS was initially performed in cereal crops, such as maize, for which flowering time was found to be associated with *Drawf8* polymorphisms^7^. In rice, GWAS was performed for 14 traits using a population consisting of 517 landraces with 3.6 million single nucleotide polymorphisms (SNPs)^8^. GWAS was also performed in rice to rapidly identify genes influencing agronomic traits using the gene-based association analysis^9^. Despite the wide applications of GWAS in cereal and other annual crops, only a few GWAS investigations have been conducted for perennial fruit trees till now. One addressed 129 peach accessions and explored 12 fruit and flower-related traits^10^. A second study explored pathogen resistance in apricot^11^. Recently, a 200K array was developed to facilitate GWAS of two traits in pear^12^. Therefore, additional GWAS with large population size, wide genetic background, and high-density SNPs are necessary to better understand the genetic architecture of fruit quality and phenological traits for fruit tree crops.

The skin color and the contents of sugars, acids, and stone cells are important pear fruit quality traits and also the main targets for pear fruit improvement. However, studies of fruit quality traits in pears are relatively limited compared with other fruit trees such as apple and grape, with only a few reports on pear fruit skin color^13^, sugar^14^, organic acid^15^, and stone cells^16,17^. Pear fruits have special brachysclereid cells called stone cells with secondary-thickened cell walls that are formed from parenchyma cells via the deposition of lignins and celluloses^18^. Recently it was determined that the lignin forms present in pear stone cells are primarily guaiacyl-lignin (G-lignin) and syringyl-lignin (S-lignin) monomers^19^.

In this study, we carry out whole-genome resequencing with an average of around ten-fold coverage on an association panel comprising 312 sand pear (*Pyrus pyrifolia*) accessions collected from China, Korea, and Japan, and revealed their population structure. Meanwhile, landrace and cultivated sand pears are explored to identify selective sweep signatures in the genome during pear improvement. We then perform GWAS of 11 agronomic traits for which phenotype data are collected over three successive years, to identify the associated genome loci and candidate functional genes. A gene involved in regulating lignin contents is identified to function in stone cell formation in pear fruits, and it is verified in both transgenic pear and *Arabidopsis*. Genome loci associated with agronomic traits of pear and the identified gene involved in pear stone cell formation provide valuable information for fruit trees breeding, and for deeper understanding of the fruit biology.

## Results and discussion

### Sequencing, variants, and population structure of sand pears

We analyzed a germplasm diversity panel comprising 312 sand pears (*P. pyrifolia*), of which 231 were landraces and 81 were improved pear cultivars (Supplementary Data 1). Genomes of these accessions were sequenced using the Illumina technology, and a total of 2.15 Tb of raw sequence data were obtained. After removing low-quality and adapter sequences, an average of about ten-fold coverage data were obtained for each accession and used for SNP calling. The average mapping rate of the cleaned reads to the reference genome of *Pyrus bretschneideri*^20^ was 73.5% (Supplementary Data 1), higher than that in a previous study of 113 pears from different species^21^.

**Fig. 1:**
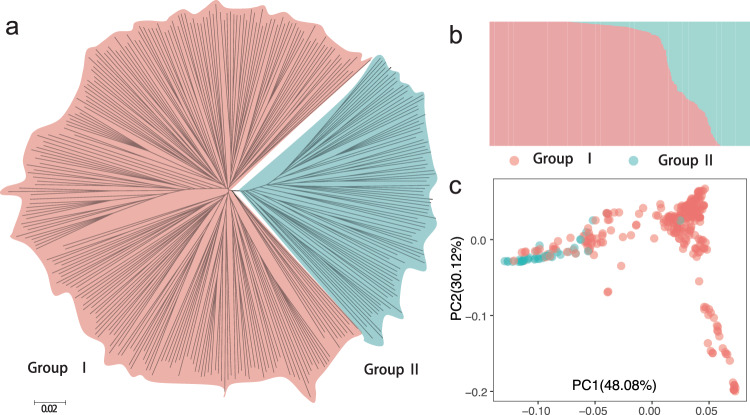
Phylogenetic relationship and population structure of 312 sand pears. **a** Phylogenetic tree of sand pears. **b** Population structure of sand pears. *K* value is 2. **c** PCA of sand pears. Group I with red background corresponds to the Chinese group, and Group II with blue background indicates the Japanese and Korea group.

A total of 3.40 million SNPs with missing rate of no more than 30% and a minor allele frequency (MAF) ≥0.03 were obtained and used for downstream analyses. We randomly selected 3942 SNP loci for Sanger sequencing, which revealed a high accuracy of the called SNPs (97.6%; Supplementary Data 2). Using these high-quality SNPs, we first inferred phylogenetic relationships of the sand pears, which suggested that the sand pears we analyzed were divided into two major subpopulations, one comprising accessions from China (Group I) and the other comprising accessions from Japan and the Korean peninsula (Group II) (Fig. 1a). This was largely consistent with the results obtained from the population structure and *F*_ST_ analysis (Fig. 1b and Supplementary Figs. 1–3) and the principal component analysis (PCA) (Fig. 1c).

### Selective sweep signals during pear improvement

**Fig. 2:**
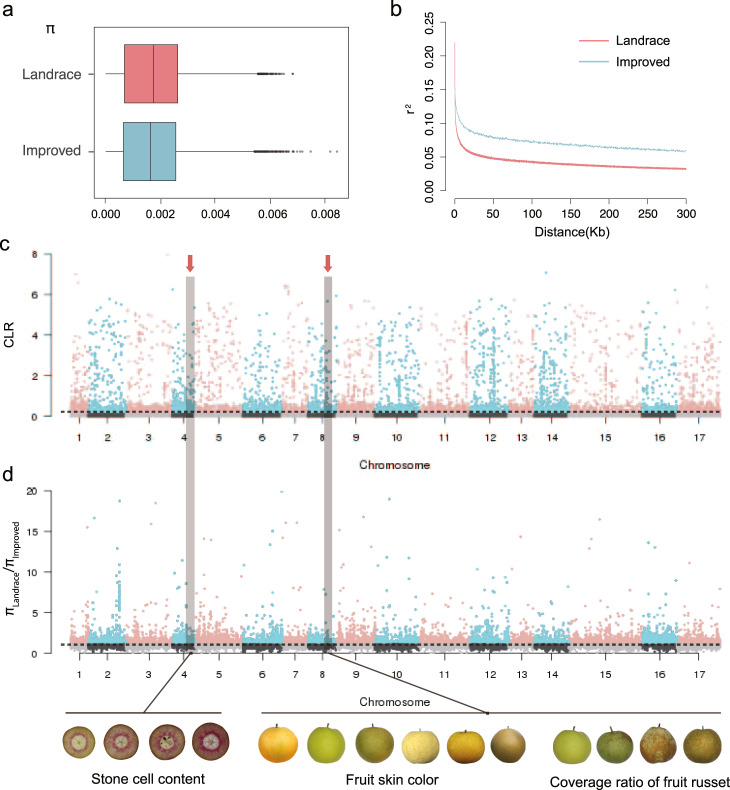
Diversity and selective sweep of landrace and improved population. **a** Nucleotide diversity (*π*) of sand pears in landrace (*n* = 231) and improved (*n* = 81) groups. For each box plot, the lower and upper bounds of the box indicate the first and third quartiles, respectively, and the center line indicates the median. The whisker represents 1.5× interquartile range of the lower or upper quartile. **b** LD in the landrace and improved groups. **c** Selective sweep regions during sand pear improvement (landrace vs improved groups) detected by CLR. The horizontal dashed line refers to the top 5% threshold of CLR scores. **d** Selective sweep regions during sand pear improvement (landrace vs improved groups) by *π* ratios. The horizontal dashed line refers to the top 15% threshold of *π*_Landrace_/*π*_Improved_. Fruit quality-related traits, stone cell content, coverage ratio of fruit russet, and fruit skin color, are demonstrated in the combined selective sweep regions.

The nucleotide diversity (*π*) of the entire sand pear population here was 1.73 × 10^−3^, and that of the landrace (1.75 × 10^−3^) was higher than that of the improved pears (1.70 × 10^−3^) (Fig. 2a). As expected, the diversity level we detected here for sand pear was substantially lower compared to that (5.5 × 10^−3^) of combined Asian and European cultivated and wild pears reported previously^21^. This previous study^21^ indicated that similar diversity was preserved in pears during the domestication process, which is different from the strong selection in annual crops, and during the improvement process, the diversity reduction in pear was slightly lower than that in annual crops such as soybean and rice^22,23^. At the transcriptome level, *π* of pear landrace group was also higher than that in the improved group, but *π* remains the same level from wild group to landrace group^24^. The linkage disequilibrium (LD) decay of the improved pears was less rapid than that of landraces (Fig. 2b). What was more, the LD decay in pears was much more rapid than that in annual crops like rice^23^ and soybean^25^, but similar to that in apple^26^, mainly due to the long generation time and self-incompatibility.

We identified candidate selective sweeps during pear improvement based on composite likelihood rates and nucleotide diversity throughout the genome for all of the accessions in the germplasm diversity panel of sand pears, which in total covered 11.1 Mb (2.89%; Supplementary Data 3) of the pear genome (Fig. 2c) and harbored 1417 genes (Supplementary Data 4), of which some were known to be related to metabolisms of sugars, organic acids, amino acids, lignin, as well as resistance and photosynthesis. We previously found that during the domestication process, genes related to metabolisms of sugars, organic acids, lignin were also under selection^21^. However, the fruit size-related genes were enriched in domestication sweeps^21^ while no such genes were found in selective sweep regions during pear improvement. We conclude that traits of lignin, sugar, and acid contents were under continuous selection during both domestication and improvement. It was also reported that lignin biosynthesis-related genes were in the selective sweep signals during domestication and improvement at the transcriptome level^24^.

### GWAS of fruit quality and phenological traits

**Fig. 3:**
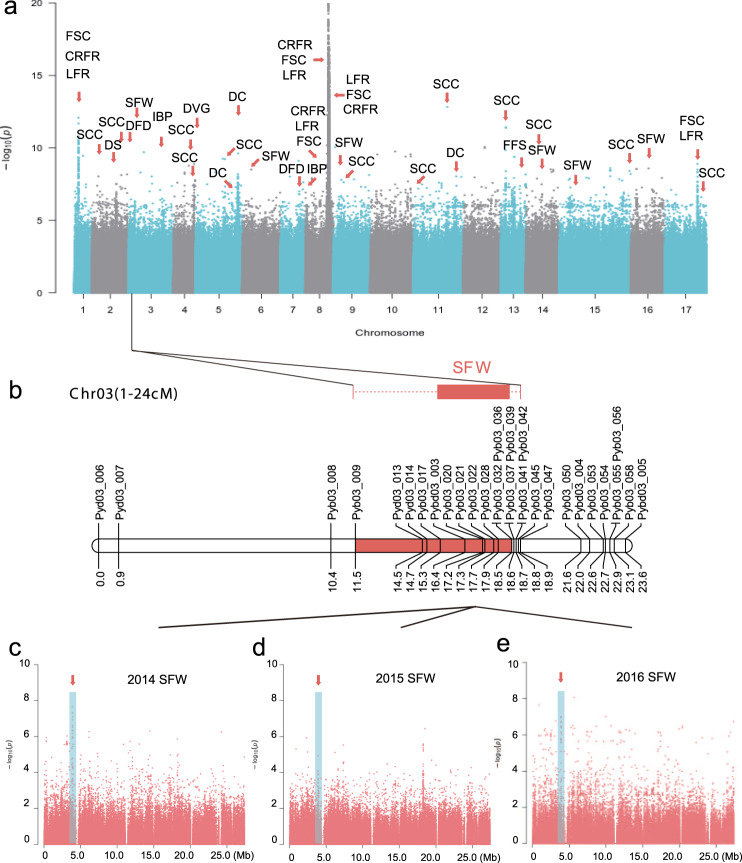
GWAS of 11 traits in 312 sand pears. **a** Manhattan plot of GWAS for 11 traits in sand pears. IBP initial bloom period, DFD days of fruit development, DVG days of vegetative growth, FSC fruit skin color, CRFR coverage ratio of fruit russet, LFR location of fruit russet, FFS furrows on fruit surface, DC direction of carpopodium, DS direction of sepal, SFW single fruit weight, SCC stone cell content. **b** QTL for trait of single fruit weight in the population of ‘Bayuehong’ × ‘Dangshansuli’. **c**–**e** Manhattan plots of chromosome 3 of GWAS for trait of single fruit weight in the years 2014 (**c**), 2015 (**d**), and 2016 (**e**).

A total of 11 agronomic traits were investigated in the 312 sand pears for three consecutive years (Supplementary Data 5 and Supplementary Fig. 4), including three fruit phenological traits (initial bloom period, days of fruit development, and days of vegetative growth) and eight fruit quality traits (single fruit weight, stone cell content, fruit skin color, coverage ratio of fruit russet, location of fruit russet, furrows on fruit surface, direction of carpopodium, and direction of sepal). We identified five candidate loci for the three phenological traits of sand pears and 37 candidate loci for the eight fruit quality traits (Fig. 3a, Supplementary Data 6, 7, and Supplementary Figs. 5–15).

Single fruit weight is very important for fruit production, and our GWAS identified five lead associated SNPs with this trait. Of particular note, one of these candidate SNPs located within a region on chromosome 3 which was previously reported to have a high LOD score for single fruit weight in a QTL study^2^ (Fig. 3b–e). Another candidate SNP (Chr9_2479073) is located within a gene (*Pbr001726*) encoding a stachyose synthase precursor, suggesting a plausible inference about the impact of this gene on starch accumulation and thus single fruit weight.

Fruit skin color has been a major focus in modern breeding programs for many fruit tree species. Our GWAS results revealed three candidate loci for this trait, and we also found that the skin color-associated loci were in regions under selection during the improvement process for sand pears (Figs. 2c, d and 3a). It was notable that eight of the candidate genes for this trait were annotated as *MYB* transcription factors, which have been widely reported to regulate fruit skin and flesh colors such as in apple^27^, strawberry^28^, kiwifruit^29^, and tomato^30^.

The furrows on fruit surface affect fruit appearance, but the molecular basis of furrow development remains poorly understood. Our GWAS of furrows on fruit surface trait identified one lead associated SNP on chromosome 13 (Chr13_11586174) (Fig. 3a and Supplementary Data 7). Furthermore, our GWAS identified one SNP (Chr8_14112990) that was significantly associated with both the coverage ratio of fruit russet and the location of fruit russet, and the associated candidate gene encoded an endoglucanase (Fig. 3a and Supplementary Data 7). The candidate region of coverage ratio of fruit russet and the location of fruit russet was in the selective region, suggesting that these two fruit russet traits could have been under selection during sand pear improvement.

The phenological trait, days of fruit development, dramatically affects fruit maturation, and the appropriate regulation of the pear fruit maturation period could substantially contribute to efficient shipping and storage of pears. Two candidate genome loci associated with this trait were identified, one of them was corresponded to the predicted gene *Pbr013897* (Fig. 3a and Supplementary Data 7), which encodes a gibberellin 20-oxidase. It has been reported that gibberellin 20-oxidase is involved in fruit growth in apple^31^, and it could also regulate the flowering and stolon differentiation in strawberry^32^.

### A stone cell-related gene from an uncharacterized family

**Fig. 4:**
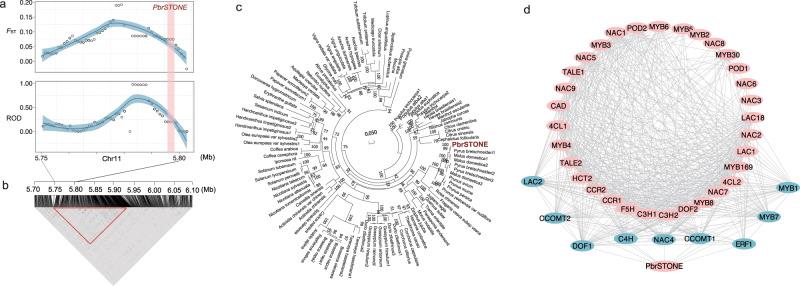
Stone cell content-related gene *PbrSTONE*. **a** Distribution of *F*_ST_ and ROD in the *PbrSTONE* gene region in landrace and improved groups. The shaded region in pink indicates the location of *PbrSTONE*, and the shade region in blue presents the smooth local regression by ‘loess’. **b** LD in the *PbrSTONE* gene region. **c** Phylogenetic tree of homologous genes of *PbrSTONE* in 81 plant species. **d** Co-expression network of *PbrSTONE* with lignin related genes. Source data underlying (**c**, **d**) are provided as a Source Data file.

High accumulation of stone cells significantly affects the quality of fruit and is particularly prevalent in sand pear fruit flesh, wherein they distribute in a mosaic pattern^33^. These brachysclereid cells have secondary thickened cell walls and ultimately become parenchyma cells with lignin and cellulose accumulation^18^. Our GWAS identified 12 candidate loci containing a total of 35 SNPs on chromosomes 2, 4, 5, 9, 11, 13, 14, 15, and 17 that were associated with stone cell content (Figs. 3a and 4a, b, and Supplementary Data 7). Since stone cells in pear are mainly produced at early stages of fruit development^16^, we next examined the fruit development RNA-Seq data that we previously generated for four pear cultivars (‘Dangshansuli’, ‘Hosui’, ‘Yali’, and ‘Starkrimson’)^20,34^ to identify genes in these loci that were highly expressed in the early fruit development. Among the identified candidate genes, in addition to previously characterized lignin regulating genes such as those encoding cinnamate 4-hydroxylases^35^, *PbrSTONE* (lignin-related stone cell formation; *Pbr005042*) gene was of particular interest. This gene showed no characterized functions from other organisms, and its encoded protein contained a conserve domain (DUF1223) with an unknown function.

We measured stone cell contents in 312 sand pear accessions, which revealed that the landrace accessions had significantly more stone cells than the improved cultivars (*p*-value = 3.69E−15) (Supplementary Fig. 16). Genome region of *PbrSTONE* had high *F*_ST_ and reduction of diversity (ROD) values indicated that it has been under selection (Fig. 4a, b). Furthermore, transcript of *PbrSTONE* was abundantly expressed at early stages of fruit development (Supplementary Fig. 17). We constructed a phylogenetic tree using *PbrSTONE* and its homologs in 81 eudicot species, and found *PbrSTONE* and its homologous genes from *Malus domestic*, *Prunus avium*, *Prunus persica*, and *Juglans regia* clustered in one group (Fig. 4c). We also carried out gene co-expression network analysis to find candidate genes from the lignin biosynthetic pathway and transcription factors correlated with *PbrSTONE*, and identified seven genes including *PbrLAC2* that had similar expression patterns with *PbrSTONE* (Fig. 4d).

**Fig. 5:**
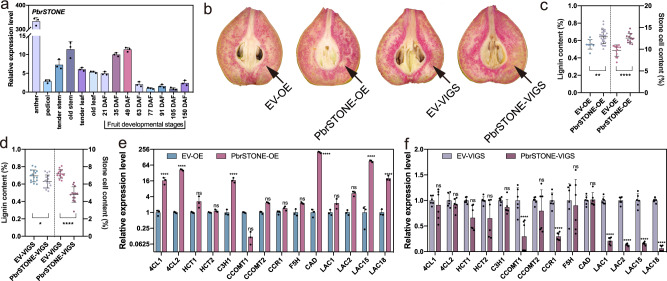
Functional validation of *PbrSTONE* in controlling stone cell content and lignin of pears. **a** Expression profiles of *PbrSTONE* in different tissues of ‘Dangshansuli’ plants determined by q-RT-PCR; *n* = 3 biologically independent samples. **b** Transient assays using *PbrSTONE* overexpression and silencing constructs in ‘Dangshansuli’ fruit at 35 days after flowering (DAF). Images were taken at 10 days after agro-infiltration. **c**, **d** Lignin and stone cell contents in the flesh tissue around the infiltration sites. More than six fruits were injected with each construct in an experiment that was repeated three times. EV empty vector, OE overexpression, VIGS virus-induced gene silencing. For lignin content, *n* = 8, 27, 16, and 16 biologically independent samples for EV-OE, *PbrSTONE*-OE, EV-VIGS, and *PbrSTONE*-VIGS, respectively. For stone cell content, *n* = 13 biologically independent samples for all lines. **e**, **f** Expression levels of genes encoding enzymes involved in monolignol biosynthesis and polymerization in the flesh tissue around the infiltration site using qPCR analysis; *n* = 3 biologically independent samples for EV-OE and *PbrSTONE*-OE, and *n* = 6 for EV-VIGS and *PbrSTONE*-VIGS. Data presented are mean ± SD; *p*-values were determined by two-tailed Student’s *t*-test (*p < 0.05, **p < 0.01, ***p < 0.001, ****p < 0.0001; ns, not significant). Source data are provided as a Source Data file.

We then conducted a qPCR-based analysis of *PbrSTONE* gene in different tissues of ‘Dangshansuli’ plants, which revealed relatively high expression levels of *PbrSTONE* in anthers, stems, and young fruits (from 21 days after flowering (DAF) to 49 DAF) (Fig. 5a), but much lower levels in pedicels and leaves, as well as in older fruits (after 63 DAF) (Fig. 5a). The expression pattern of *PbrSTONE* during pear fruit development was consistent with the fact that lignin deposition in stone cells generally occurs during the early stages of fruit development^16^.

To further elucidate the functional role of *PbrSTONE* in lignin biosynthesis of stone cells, the *PbrSTONE* overexpression or silencing constructs were agro-infiltrated into ‘Dangshansuli’ pear fruit at 35 DAF. We confirmed the expression of *PbrSTONE* (Supplementary Fig. 18a) at 10 days post infiltration, and also observed a dramatic change of the lignin staining signal at the infiltration sites. Overexpressing *PbrSTONE* significantly increased stone cell and lignin contents, while silencing *PbrSTONE* decreased them (Fig. 5b–d). The most striking observation from these transient expression experiments was that regulating *PbrSTONE* expression caused a significant change of the expression of lignin biosynthesis-related genes, particularly the expression of *LAC*s whose homologous genes in *Arabidopsis* are known to be involved in lignin polymerization^36,37^ (Fig. 5e, f).

**Fig. 6:**
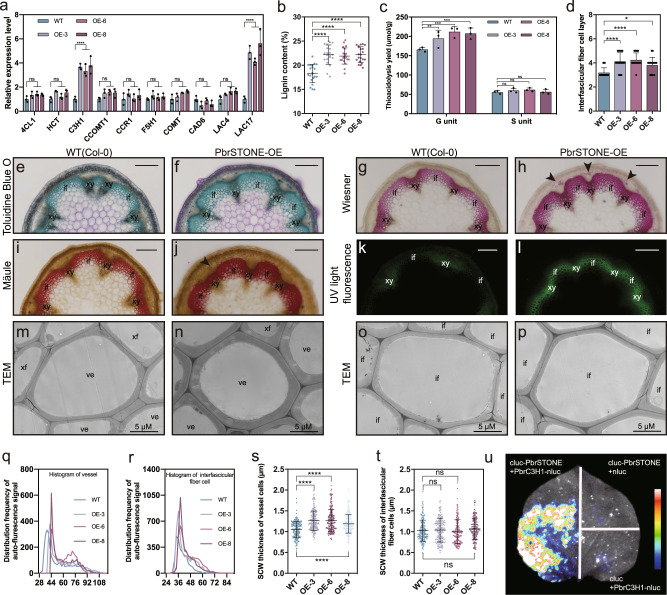
Functional validation of *PbrSTONE* in *Arabidopsis*. **a** Expression levels of genes encoding enzymes involved in monolignol biosynthesis and polymerization in WT and *PbrSTONE* transgenic *Arabidopsis* plants using qPCR analysis; *n* = 3 biologically independent samples. **b**, **c** Lignin analysis for WT and *PbrSTONE* transgenic *Arabidopsis* plants; h: *n* = 21 biologically independent samples for **b**, and *n* = 3 for **c**. **d** Layers of stem interfascicular fiber cells in transgenic *Arabidopsis* plants expressing *PbrSTONE*; *n* = 16 biologically independent samples. **e**, **f** Cross-sections of 8-week-old inflorescence stems stained with Toluidine blue O. **g–j** Cross-sections of 8-week-old inflorescence stems stained with Mäule and Wiesner reagent, which bind to S-lignin monomer and G-lignin monomer, respectively. **k**, **l** Lignin auto-fluorescence of cross-sections detected under UV light. Scale bars: 200  μm in **k**–**r**. Bar in **k–r** = 200 μm; xy, xylem; if, interfascicular fiber cell. The transgenic and WT plants were cultivated two times and cross-sections were taken from five independent plants which showed similar results. A representative picture from each line is shown. **m**–**p** Transmission electron micrographs of the cross-section of cells in **e**; ve, vessel; xf, xylary fiber; if, interfascicular fiber cells. **q**, **r** Statistical analysis of lignin auto-fluorescence signal in the vessel cells and interfascicular fiber cells. More than 12 samples in each line were analyzed. **s**, **t** Statistical analysis of secondary cell wall thickness in the vessel cells and interfascicular fiber cells. More than 50 cells in each line were analyzed. **u** Firefly luciferase complementation assay in young *Nicotiana benthamiana* leaves. Data presented are mean ± SD; *p*-values were determined by two-tailed Student’s *t*-test (*p < 0.05, **p < 0.01, ***p < 0.001, ****p < 0.0001; ns, not significant). Source data underlying (**a**–**t**) are provided as a Source Data file.

We next generated transgenic *Arabidopsis* lines stably expressing *PbrSTONE* under the control of the 35S promoter. We confirmed the constitutive expression of *PbrSTONE* in stems of 4-week-old plants from three overexpression lines (*PbrSTONE-OE*) (Supplementary Fig. 18b). In T_3_ generation homozygous plants, qPCR analysis of 4-week-old wild-type (WT) and *PbrSTONE-OE* plants revealed that the expression of the *Arabidopsis* homologs of the pear lignin biosynthesis genes *PbrC3H1* (*AtC3H1*) and *PbrLAC15/18* (*AtLAC17*) were significantly induced by transgenic overexpression of *PbrSTONE* (Fig. 6a). We analyzed a variety of phenotypes and found several major differences between WT and *PbrSTONE-OE* plants. Specifically, although the final height and diameter of the primary inflorescence stem of the transgenic lines did not differ from the WT at 8 weeks (Supplementary Fig. 19), the transgenic plants had significantly increased lignin content and decreased root length compared to the WT (Fig. 6b and Supplementary Fig. 19). Moreover, the transgenic plants had significantly increased accumulation of G-lignin monomers compared to WT plants, whereas no differences were observed for the accumulation of S-lignin monomers (Fig. 6c).

Toluidine blue O staining showed that the *PbrSTONE-OE* stems contained significantly higher numbers of interfascicular fiber (IF) cell layers than WT plants (Fig. 6d–f). Both the vessels and IF cell tissues of the *PbrSTONE-OE* lines exhibited stronger chemical staining and auto-fluorescence signals for lignin components than did the WT tissues (Fig. 6g–l, q, r), further supporting that *PbrSTONE* expression dramatically alters lignin metabolism. These results support that *PbrSTONE* functions in both IF formation and lignin deposition in vessels and in IF cell tissues.

To examine anatomical features of the secondary cell walls of the multiple *PbrSTONE-OE* lines, we conducted transmission electron microscopy (TEM) analysis. We observed that the secondary cell walls of the vessels in *PbrSTONE-OE* lines were significantly thicker than in WT plants, whereas no such difference in secondary cell wall thickness was observed when examining the IF tissue (Fig. 6m–p, s, t).

In order to better understand how *PbrSTONE* regulates lignin formation in stone cells, BiFC-LUC assay was conducted with candidate co-expressed genes to identify potential PbrSTONE interacting proteins. PbrSTONE was found to interact with PbrC3H1 (Fig. 6u), which was positively regulated by a positive regulator of lignification and stone cell development, PbrMYB169 (ref. ^17^). The homologous gene of *PbrC3H1* in *Arabidopsis*, *AtREF8* (AT2G40890.1), encodes a coumarate 3-hydroxylase (C3H), a P450-dependent monooxygenase, and is involved in lignin and flavonoid biosynthesis^38^. *PbrSTONE* was cytoplasm and cytomembrane localized as determined by PbrSTONE-GFP fusion protein in transgenic *Arabidopsis* root (Supplementary Fig. 20), consistent with the previously reported subcellular localization of C3H^39^. Taken together, these results suggested that PbrSTONE may take part in lignification of stone cells via interacting with PbrC3H1 to form a protein complex.

## Methods

### Agronomic trait measurement

All of the measurements for the agronomic traits were based on the Description and data standard for pear (*Pyrus spp*.)^40^. Data were collected in 2014, 2015, and 2016 (some traits were recorded in 2 years because of the unpredictable dropped fruits). Single fruit weight was recorded as the average weight of ten fruits from each sample. Fruit skin color was recorded as ‘1’ to ‘6’ according to the Supplementary Fig. 21. Coverage ratio of fruit russet was defined as the ratio of russet area to fruit skin area in the mature fruits. Data were recorded as ‘1’, ‘3’, ‘5’, ‘7’ (Supplementary Fig. 22). Location of fruit russet was recorded as ‘1’, ‘2’, ‘3’, and ‘4’ (Supplementary Fig. 23). Furrows on mature fruit surface were recorded as ‘0’ and ‘1’ (Supplementary Fig. 24). Direction of carpopodium was recorded as ‘1’ and ‘2’ according to the Supplementary Fig. 25. Direction of sepal was recorded as ‘1’, ‘2’, and ‘3’ according to the Supplementary Fig. 26. For days of fruit development, days of vegetative growth, and initial bloom period, data were recorded as the calendar date and were calculated as the number of days from the earliest date to the last one. The stone cell contents were measured according to Tao et al.^33^. Briefly, pulp of mature fruits was collected, stored at −20 °C for 24 h, and then homogenated with juice extractor. The sample was centrifuged, and after centrifugation the supernatant was removed. The resulting pellets (stone cells) were re-suspended in water, vortexed, and centrifuged. These washing steps were repeated several times until the supernatant was transparent. The collected stone cells were oven-dried and then weighed three times.

### Genome re-sequencing

Young leaves from a total of 312 sand pear accessions (Supplementary Data 1) were collected from the Wuchang Sand Pear Germplasm collection (Hubei Academy of Agricultural Sciences, China). DNA was extracted using plant genomic DNA kits from TIANGEN^®^. DNA quality was checked by NanoPhotometer^®^ spectrophotometer (IMPLEN) and 1% agarose gels, and DNA concentration was measured by Qubit^®^ DNA Assay Kit in a Qubit 2.0 Flurometer (Life Technologies). NEB Next Ultra DNA Library Prep Kit for Illumina^®^ (NEB) was used to generate the sequencing libraries. The cBot Cluster Generation System of the HiSeq 4000 PE Cluster Kit (Illumina) was used for clustering of the index-coded libraries, and the Illumina HiSeq 4000 system was used to sequence the DNA libraries (150 bp paired-end reads).

### Data filtering and SNP calling

Quality of the 150-bp paired-end reads was checked by FastQC (http://www.bioinformatics.babraham.ac.uk/projects/fastqc/), and the reads were then processed using Trimmomatic^41^ to trim the adapters and sequences with average quality per base below 20. The trimmed reads with length of at least 40 bp were mapped against the reference pear genome^20^ (*P. bretschneideri*) using BWA aln^42^ with parameters ‘-n 0.04 -o 1 -e 2’. Genome Analysis Toolkit (4.1.4) was used for SNP calling with unified genotyper method^43^, and the SNPs were filtered using criteria of MAF ≥0.03 and missing rate no more than 30%.

### *F*_ST_ and ROD analysis and selective sweep detection

*F*_ST_ and ROD were calculated using vcftools^44^ with a window size of 10 kb and a window step size of 5 kb, and ‘gplots’ (https://cran.r-project.org/web/packages/gplots) in the R packages was applied for presentation. RNA-Seq data of landrace and improved pears^24^ were used for *PbrSTONE* selection analysis. Candidate selective sweeps were first detected using the SweeD’s composite likelihood rate (CLR) test of the site frequency spectra^45^ with a window size of 10 kb, windows with top 5% CLR scores were considered candidate regions under a selective sweep. These regions were further filtered using *π* ratios, and only those having top 15% *π* ratios were considered the final selective sweeps. GWAS associated loci within 550 bp up or downstream of the identified selective sweeps were extracted. We also extracted genes in the selective sweep regions based on the gene annotation of the ‘Dangshansuli’ genome covered by sweeps.

### Genome-wide association studies

The pear population structure was calculated by STRUCTURE^46^ with the simulated population structure (K value) ranging from 2 to 6. GWAS were performed using both TASSEL^47^ and EMMAX programs^48^. Candidate associated loci identified by both TASSEL and EMMAX were considered further in our study, as well as those identified in one program but with genes annotated as known to be related to the corresponding traits. TASSEL was also used for the phylogenetic tree (Neighbor-Joining method), PCA, kinship matrix of the population, and LD analyses. General linear model (GLM) and mixed linear model (MLM) used for GWAS analyses were compared based on the Q–Q plots, and we finally found MLM-PCA-kinship was the best for our sand pear population. The *R*^2^ of LD was visualized using the LDheatmap R package^49^. The cutoff was used as *P* = 1E−5. The genomic inflation factor was calculated by R package of GenABEL^50^ with regression method.

### Gene annotation and statistical analysis

We used the annotated information of pear genes from KEGG and Swissport database. Specifically, the DNA and amino acid sequences of *PbrSTONE* were used for BLAST analysis against the NCBI nucleotide, protein and conserved domain databases. We also used hidden Markov model analysis to compare PrbSTONE amino acid sequences to the Pfam domain database^51^. We used Student’s *t*-tests or one-way ANOVA to compare statistical differences using GraphPad prism 8.0 software (GraphPad Software).

### Gene co-expression analysis and phylogeny of *PbrSTONE*

Genes (*4CL1*, *4CL2*, *C3H1*, *C3H2*, *C4H*, *CAD*, *CCOMT1*, *CCOMT2*, *CCR1*, *CCR2*, *F5H*, *HCT1*, *HCT2*, *POD1*, *POD2*, *LAC1*, *LAC2*, and *LAC18*) from lignin biosynthetic pathway and transcription factors (*LAC1*, *LAC2*, *LAC18*, *MYB1-8*, *MYB24*, *MYB30*, *MYB169*, *NAC1-9*, *ERF1*, *DOF1*, *DOF2*, *TALE1*, and *TALE2*) were selected as the candidate genes for co-expression network analysis with *PbrSTONE* using WGCNA^52^, using the fruit gene expression data of six developmental stages of five pear subspecies from our previous study^35^. The correlation relationship was visualized using Cytoscape (http://www.cytoscape.org).

We performed ‘blastp’ of the *PbrSTONE* protein sequence against the NCBI nr database, and obtained 99 proteins from 81 eudicot species with an e-value <1E−5 and sequence identity above 60%. All of the identified proteins were aligned with CLUSTAL^53^ (version 2.1), and the Maximum Likelihood method in IQ-TREE^54^ (multicore version 1.6.10) was applied for the phylogenic tree construction with 1000 bootstraps.

### Gene expression analysis using qPCR

cDNA was synthesized using one-step gDNA removal and cDNA synthesis kit (Transgen). qPCR was performed using the LightCycler 480 SYBR GREEN Master system (Roche). Primers are shown in Supplementary Data 8. The analysis was conducted using three biological and three technical repeats. Relative expression levels of each gene were calculated using the 2^−ΔΔCp^ method. *PbrGAPDH* and *Atactin/AtEF1α* were used as reference genes for pear and *Arabidopsis*, respectively.

### Transient transformation of *PbrSTONE* in pear fruit flesh

To transiently overexpress *PbrSTONE*, the full-length coding sequence of *PbrSTONE* was fused in frame to the N-terminus of GFP under the control of CaMV 35S promoter in the binary vector pCAMBIA1302 (p1302) to form the fusion vector 35S::*PbrSTONE*-GFP. For TRV virus-induced gene silencing (VIGS), the partial coding sequences of *PbrSTONE* (1–276 bp) was amplified using primers listed in Supplementary Data 8 and the amplified fragment was inserted into the vector TRV2. The fusion constructs and the control vector (p1300) were transferred into *Agrobacterium tumefaciens* strain GV3101 by the freeze–thaw method. For transiently transformed pear fruit flesh, the cells were infiltrated into ‘Dangshansuli’ fruit flesh at 35 DAF using needleless syringes. The transformed fruits were placed in the dark at 22 °C overnight and then incubated in a growth chamber at 22 °C under 16 h photoperiod for 10 days before being examined and imaged^16^. Six fruits were injected with each construct in an experiment that was repeated three times independently. The acetyl bromide-based method was used to detect lignin in fruit flesh tissue. The lignin content was shown as a percentage (calculated lignin content/ dry weight of stone cells × 100%)^16^.

### Arabidopsis transformation

*Agrobacterium tumefaciens* containing the vector 35S::*PbrSTONE*-GFP were transformed into *Arabidopsis* Col-0 plants using using the floral dip method^55^. Expression levels of *PbrSTONE* in primary inflorescence stem of two-week-old T_1_ plants were analyzed by semi-quantitative RT-PCR using primers listed in Supplementary Data 8. Relatively high levels of *PbrSTONE* expression were measured in three transgenic lines (OE-3, OE-6, and OE-8), which were selected to generate T_3_ homozygous plants for the subsequent analyses. Root length of each transgenic line and the WT control were measured at 7 days after germination on agar medium. Subsequently the plants were transplanted in a glasshouse with a 16 h photoperiod. The height and diameter of primary inflorescence stem were determined for each transgenic line and the WT control at 8 weeks post germination.

### Lignin analysis

For WT and T_3_ transgenic *Arabidopsis* plants, the basal 10 cm of the primary inflorescence stem was chopped and pooled together from five plants. Samples of stem pieces were extracted sequentially for water, ethanol, chloroform, and acetone to gain purified cell wall residue, then dried for the subsequent lignin analysis. Acetyl bromide-soluble lignin content was estimated according to a standard procedure^56^. The lignin composition was determined by thioacidolysis^57^. The released lignin monomers were derivatized with *N*,*O*-bis (trimethylsilyl) acetamide and quantified by GC-MS as their trimethylsilylated derivatives. The analytical data were presented as the mean values obtained from the same amount of starting material harvested from the pooled xylem tissues of five plants.

### Microscopy

For light microscopy analysis, the basal 1 cm of primary inflorescence stems of 8-week-old *Arabidopsis* WT and *PbrSTONE* transgenic plants were harvested and fixed in formalin-acetic acid-alcohol fixative (1.9% formaldehyde solution [v/v], 63% ethyl alcohol [v/v], 5% glacial acetic acid [v/v], 30.1% water [v/v]) at 4 °C for 7 days. Cross sections (100 µm thickness) of inflorescence stems were sectioned with a Leica VT1000S vibratome (Leica Mikrosysteme, Germany), stained with Toluidine Blue O, Mäule and Wiesner reagent separately, and then observed with Nikon Ni-U microscope (Nikon, Japan)^58^. Nikon Ti-E microscope (Nikon, Japan) was used to visualize the lignin auto-fluorescence under UV light (excitation at 355/25). Fluorescence intensity was measured on the images with ImageJ software.

TEM analyses were performed with the stem samples that were fixed in a fixative (2.5% [v/v] glutaraldehyde, 50 mm sodium phosphate [pH 7.2], and 4% [v/v] formaldehyde) for 12 h and further fixed by 0.5% osmic acid^59^. The tissues were then dehydrated and embedded in LR white resin (Polysciences, Warrington, PA, USA). Ultrathin sections were stained with uranyl acetate and lead citrate. The secondary wall thickness of vessel cells was gained on the images of TEM with ImageJ.

### Bimolecular fluorescence complementation analysis

pCAMBIA1300-NLuc and pCAMBIA1300-CLuc were used for bimolecular fluorescence complementation (BiFC) analysis^60^. Full-length coding sequences of *PbrSTONE* and *PbrC3H1* were amplified using the primers listed in Supplementary Data 8 and then inserted individually into the two plasmids and transferred into *A. tumefaciens* strains GV3101. Equal amounts of *PbrSTONE* and *PbrC3H1* combinations were co-infiltrated into the young leaves of *Nicotiana benthamiana* plants. On the second day post-infiltration, 1 mM luciferin was sprayed onto the leaves, and the samples were incubated in the dark for 5–10 min. LUC images were captured using a low light cooled CCD imaging apparatus (Tanon 5200 Multi).

### Reporting summary

Further information on research design is available in the Nature Research Reporting Summary linked to this article.

## Supplementary information

Supplementary Information

Reporting Summary

Description of Additional Supplementary Files

Supplementary Data 1-8

## Data Availability

Data supporting the findings of this work are available within the paper and its Supplementary Information files. A reporting summary for this Article is available as a Supplementary Information file. The datasets and plant materials generated and analyzed during the current study are available from the corresponding author upon request. Raw genome resequencing reads have been deposited into the NCBI sequence read archive (SRA) under BioProject accession PRJNA563813. Sequence data of *PbrSTONE* and *PbrC3H1* can be found at GenBank with the accession numbers MT711883 and MT711884, respectively. SNPs identified in this study are available at Zenodo (https://zenodo.org/deposit/3971245). Figures 1, 2, 3a, 3c, 3d, 3e and 4b are generated from the SNPs data using parameters specified in ‘Methods’.  Source data are provided with this paper.

## Source data

Source data
