# Supplementary material for: Genome-wide association studies provide insights into the genetic determination of fruit traits of pear
Source: Nat Commun. 2021 Feb 18;12:1144. doi: 10.1038/s41467-021-21378-y (PMC7892570; doi:10.1038/s41467-021-21378-y)
Supplement: Supplementary file 1 — Supplementary Information [file 41467_2021_21378_MOESM1_ESM.pdf]

Genome-wide association studies provide insights into the genetic determination of fruit traits of pear

Zhang *et al.*

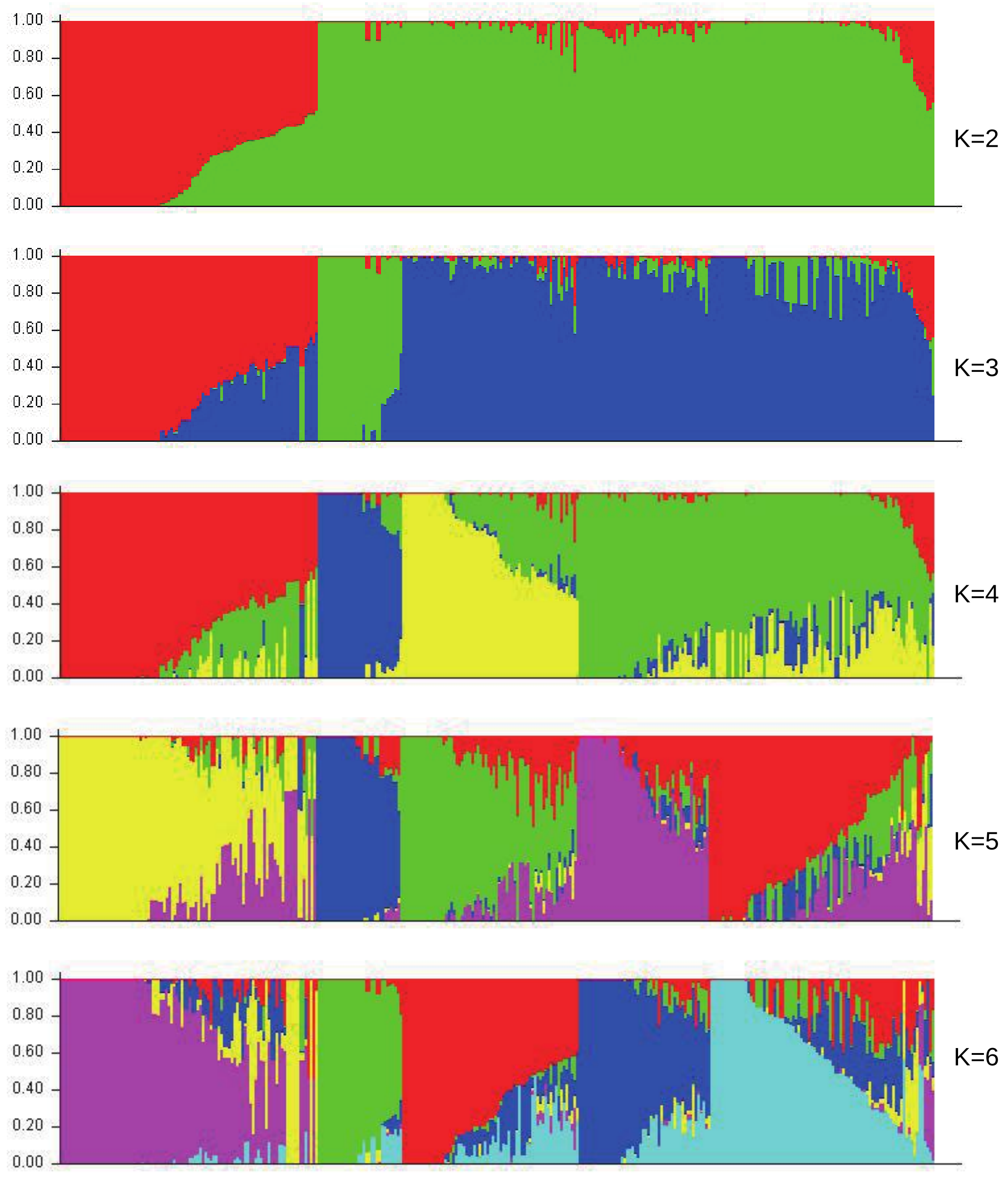

Supplementary Figure 1. Population structure of 312 sand pears with *K* values ranging from 2 to 6. Source data are provided as a Source Data file.

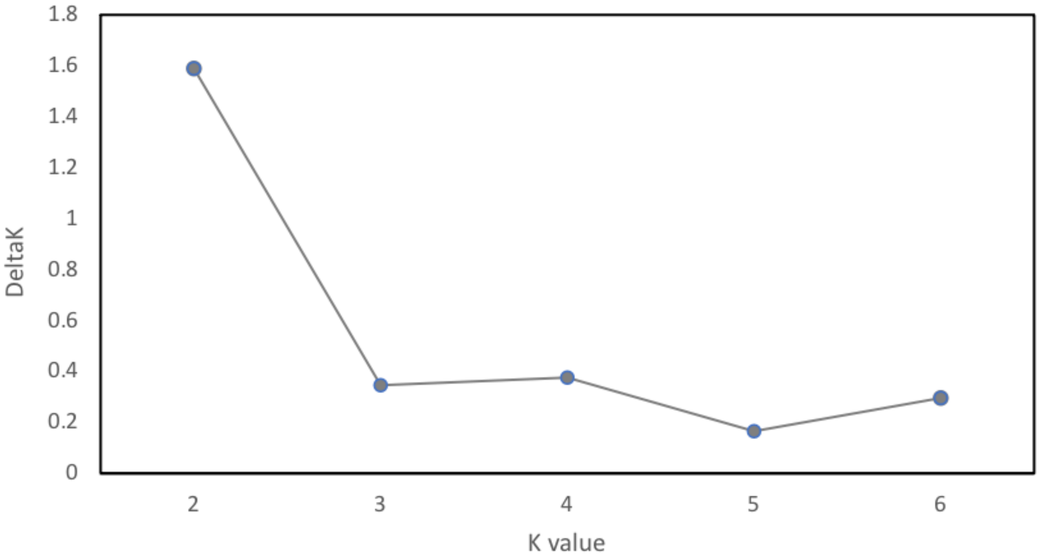

Supplementary Figure 2. DeltaK calculated with *K* from 2 to 6.

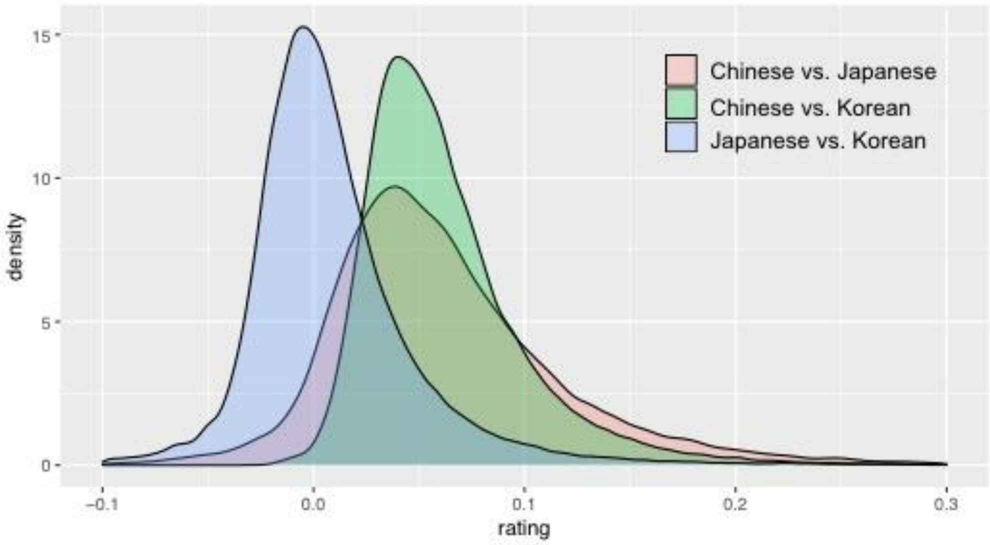

Supplementary Figure 3. F_{ST} between sand pears from different regions.

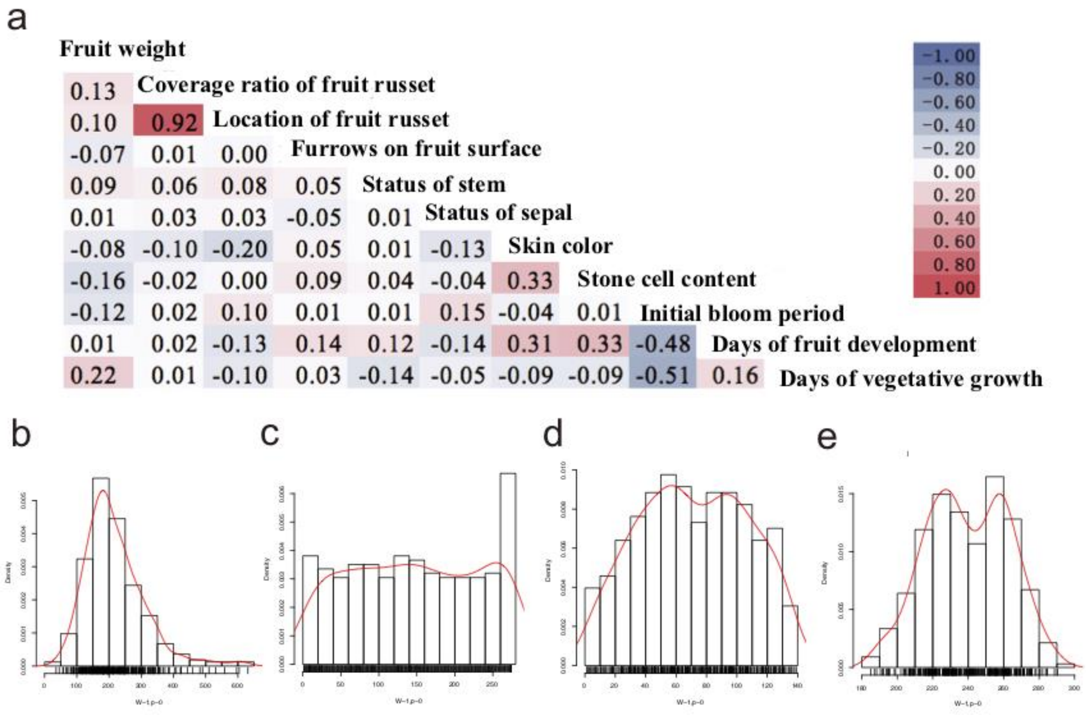

Supplementary Figure 4. Correlation and distribution of phenotypes in sand pears. **a**. Correlation of all the traits in sand pears. Values indicated correlation coefficients (*r*) between two traits. **b**. Distribution of single fruit weight in 312 sand pears. **c**. Distribution of stone cell content. **d**. Distribution of days of fruit development. e. Distribution of days of vegetative growth. Source data are provided as a Source Data file.

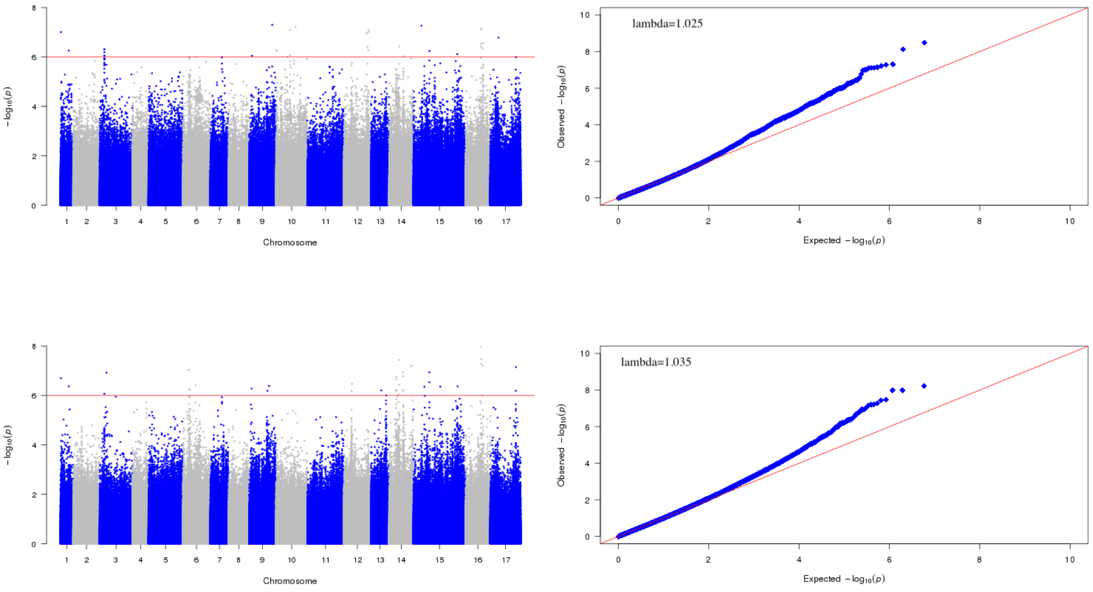

Supplementary Figure 5. Manhattan and Quantile-Quantile plot of GWAS for single fruit weight. Figures on the top and the bottom are Manhattan plots and Quantile-Quantile plots generated using TASSEL and EMMAX, respectively.

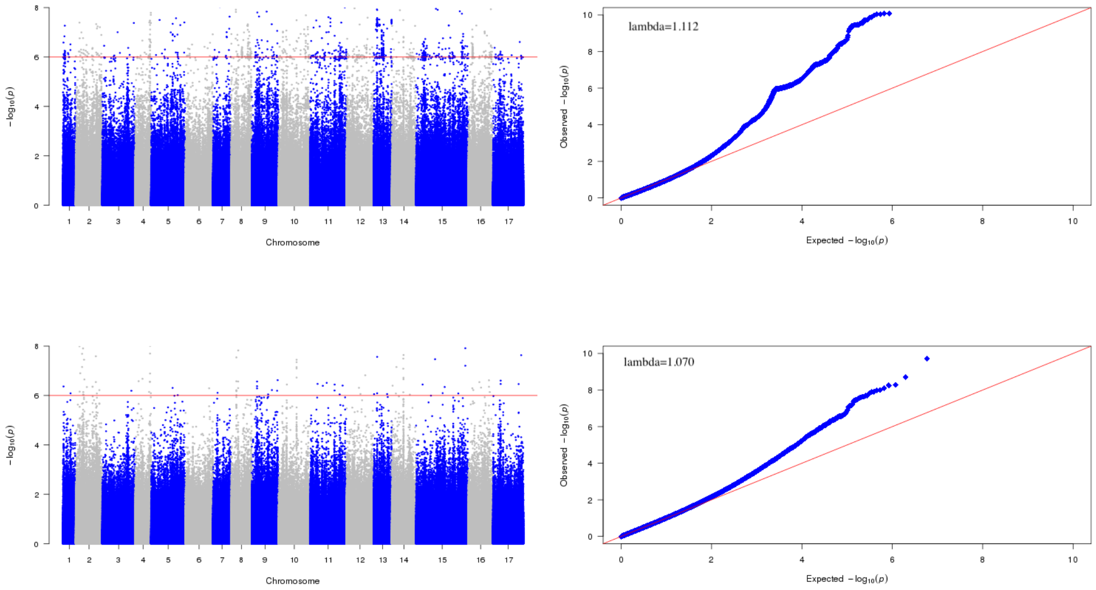

Supplementary Figure 6. GWAS and Quantile-Quantile plot of stone cells content. Figures on the top and the bottom are Manhattan plots and Quantile-Quantile plots generated using TASSEL and EMMAX, respectively.

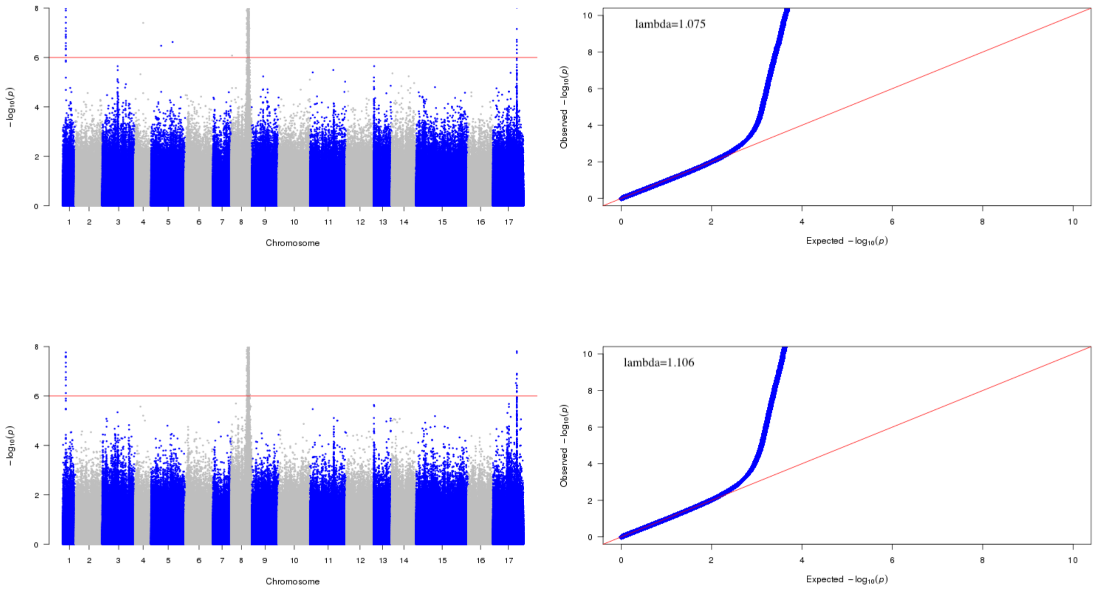

Supplementary Figure 7. GWAS and Quantile-Quantile plot of pear fruit skin color. Figures on the top and the bottom are Manhattan plots and Quantile-Quantile plots generated using TASSEL and EMMAX, respectively.

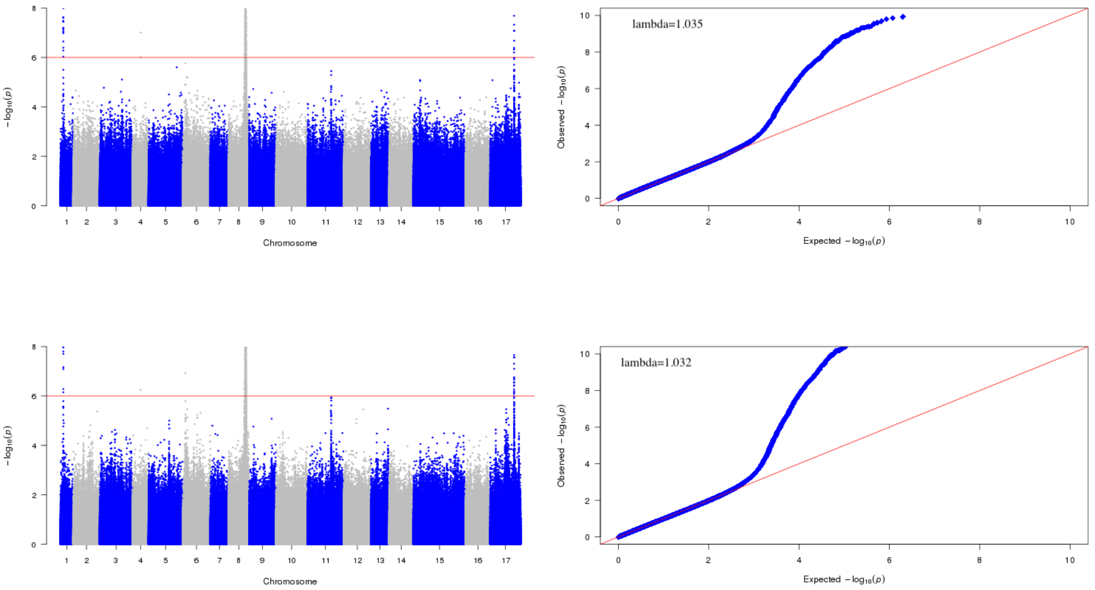

Supplementary Figure 8. GWAS and Quantile-Quantile plot of coverage ratio of fruit russet. Figures on the top and the bottom are Manhattan plots and Quantile-Quantile plots generated using TASSEL and EMMAX, respectively.

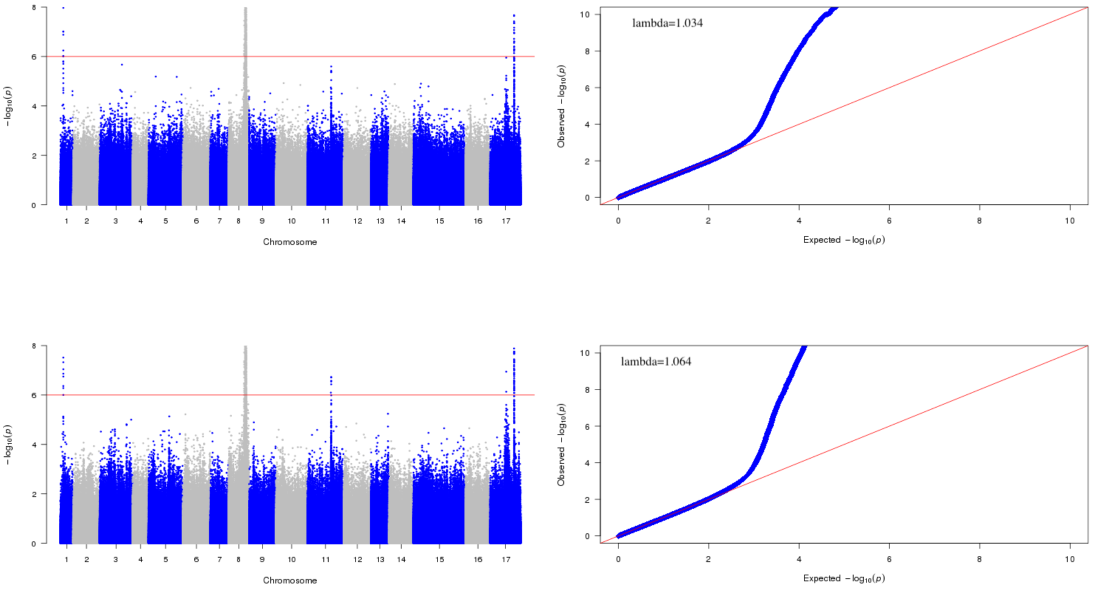

Supplementary Figure 9. GWAS and Quantile-Quantile plot of location of fruit russet. Figures on the top and the bottom are Manhattan plots and Quantile-Quantile plots generated using TASSEL and EMMAX, respectively.

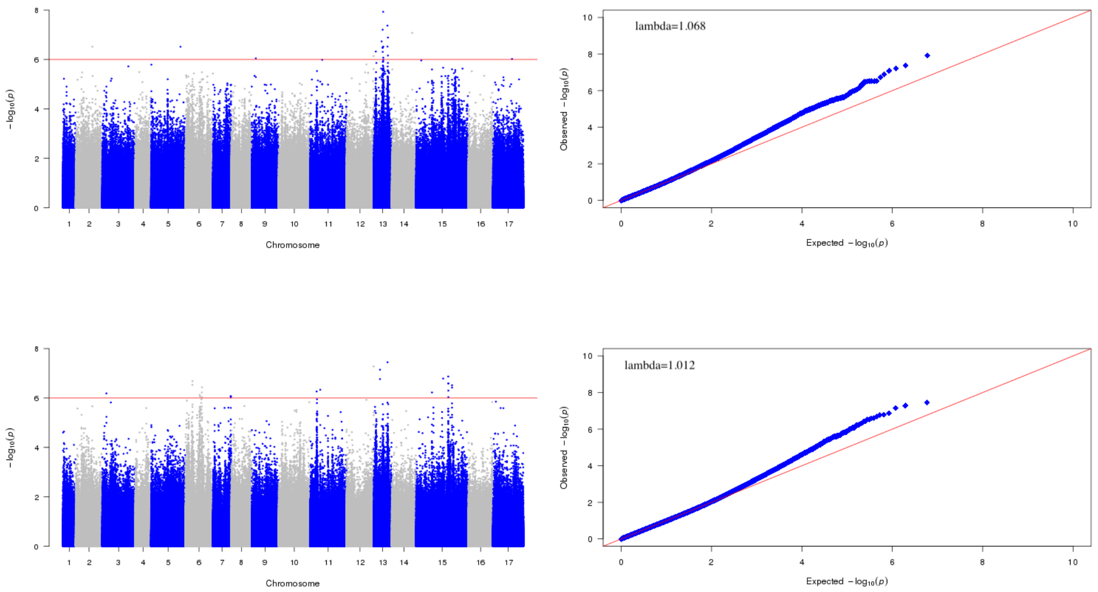

Supplementary Figure 10. GWAS and Quantile-Quantile plot of furrows on fruit surface. Figures on the top and the bottom are Manhattan plots and Quantile-Quantile plots generated using TASSEL and EMMAX, respectively.

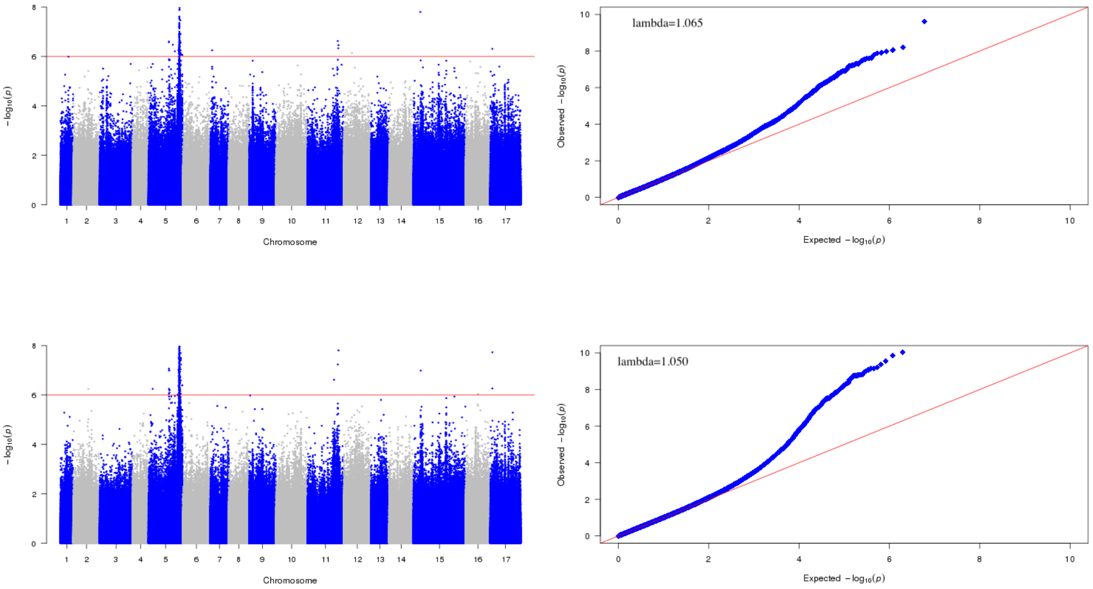

Supplementary Figure 11. GWAS and Quantile-Quantile plot for direction of carpopodium. Figures on the top and the bottom are Manhattan plots and Quantile-Quantile plots generated using TASSEL and EMMAX, respectively.

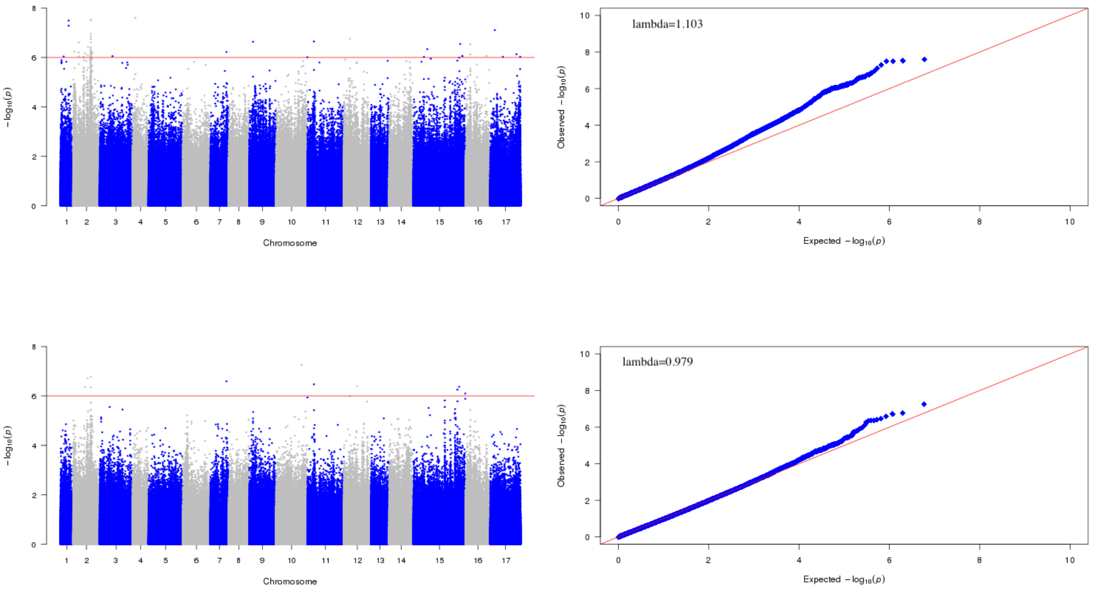

Supplementary Figure 12. GWAS and Quantile-Quantile plot for direction of sepal. Figures on the top and the bottom are Manhattan plots and Quantile-Quantile plots generated using TASSEL and EMMAX, respectively.

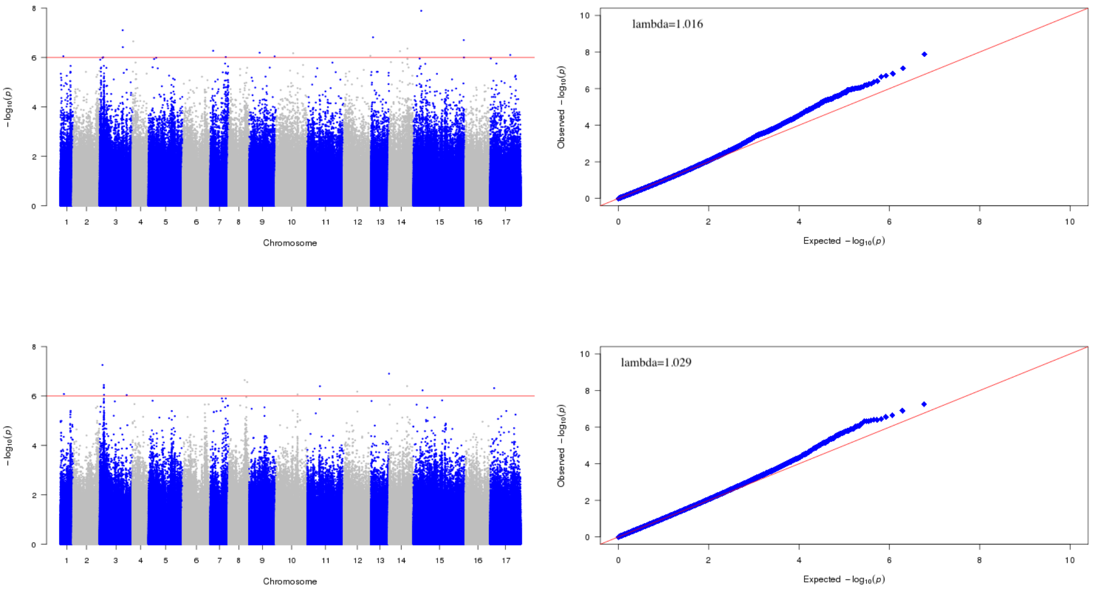

Supplementary Figure 13. GWAS and Quantile-Quantile plot for days of fruit development. Figures on the top and the bottom were Manhattan plots and Quantile-Quantile plots generated using TASSEL and EMMAX, respectively.

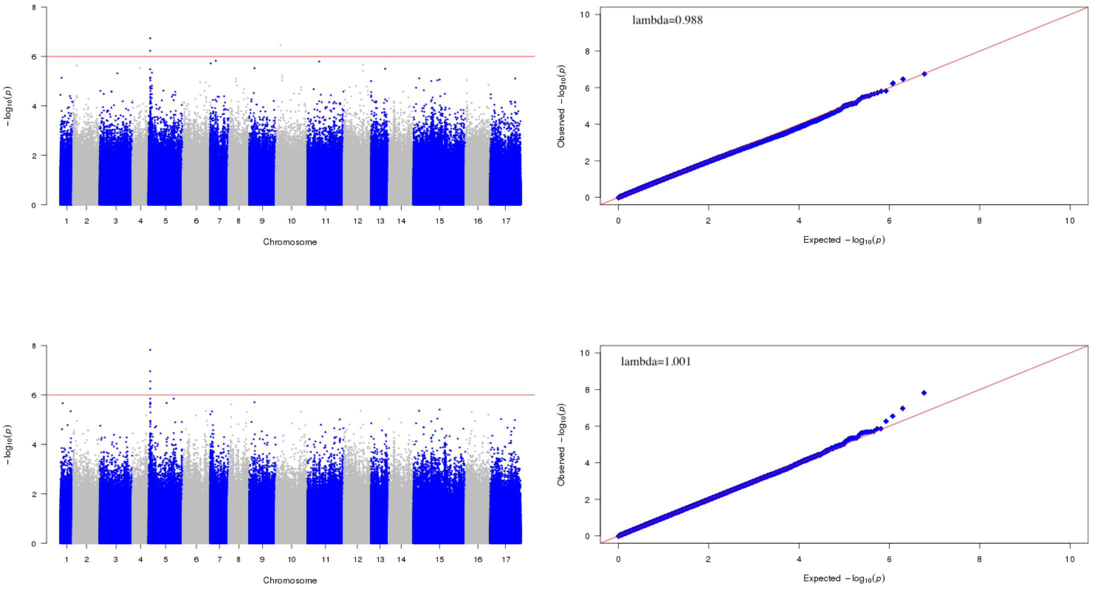

Supplementary Figure 14. GWAS and Quantile-Quantile plot for days of vegetative growth. Figures on the top and the bottom are Manhattan plots and Quantile-Quantile plots generated using TASSEL and EMMAX, respectively.

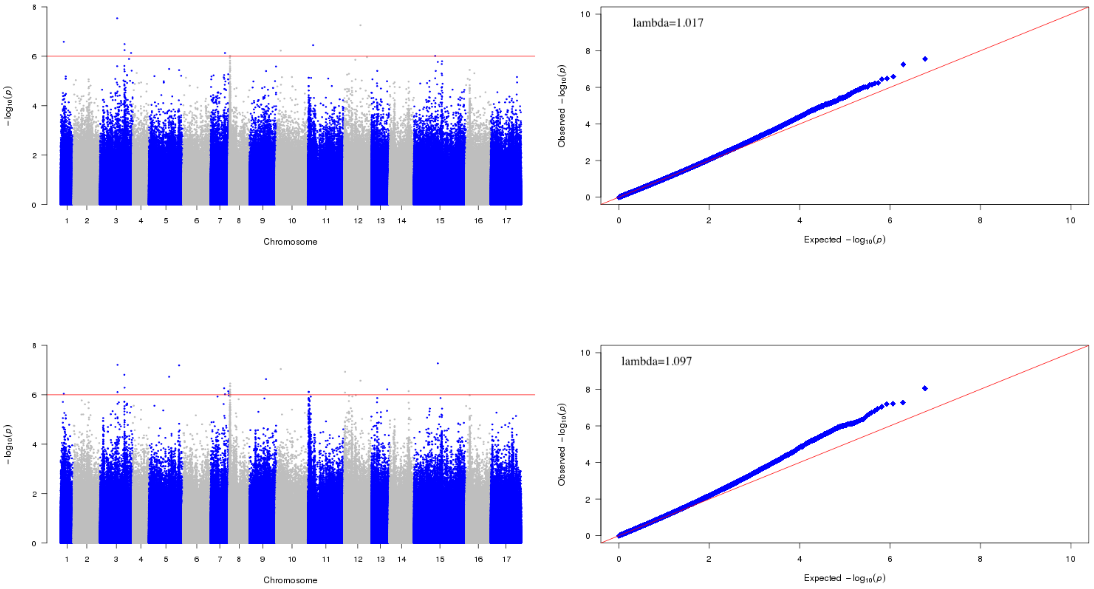

Supplementary Figure 15. GWAS and Quantile-Quantile plot of initial bloom period. Figures on the top and the bottom are Manhattan plots and Quantile-Quantile plots generated using TASSEL and EMMAX, respectively.

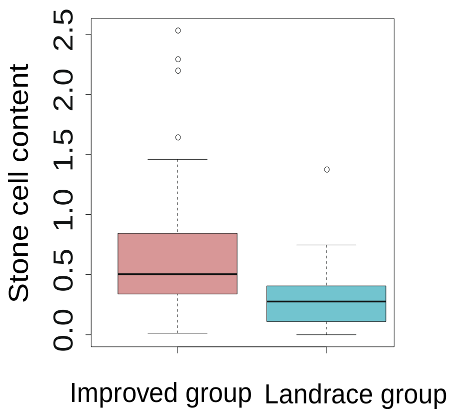

Supplementary Figure 16. Stone cell contents in landrace (n=231) and improved (n=81) groups. For each box plot, the lower and upper bounds of the box indicate the first and third quartiles, respectively, and the center line indicates the median. The whisker represents 1.5× interquartile range of the lower or upper quartile.

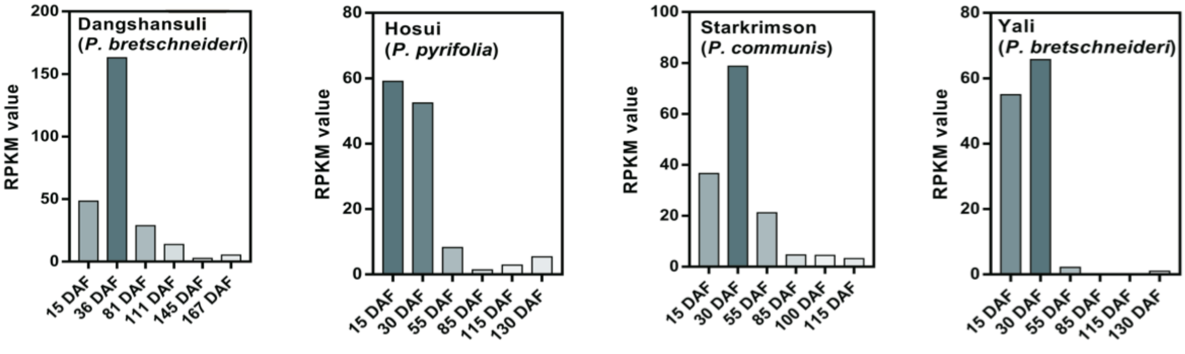

Supplementary Figure 17. Expression profiles of *PbrSTONE* during fruit development in four pear cultivars (‘Dangshansuli’, ‘Hosui’, ‘Starkrimson’, and ‘Yali’). Source data are provided as a Source Data file.

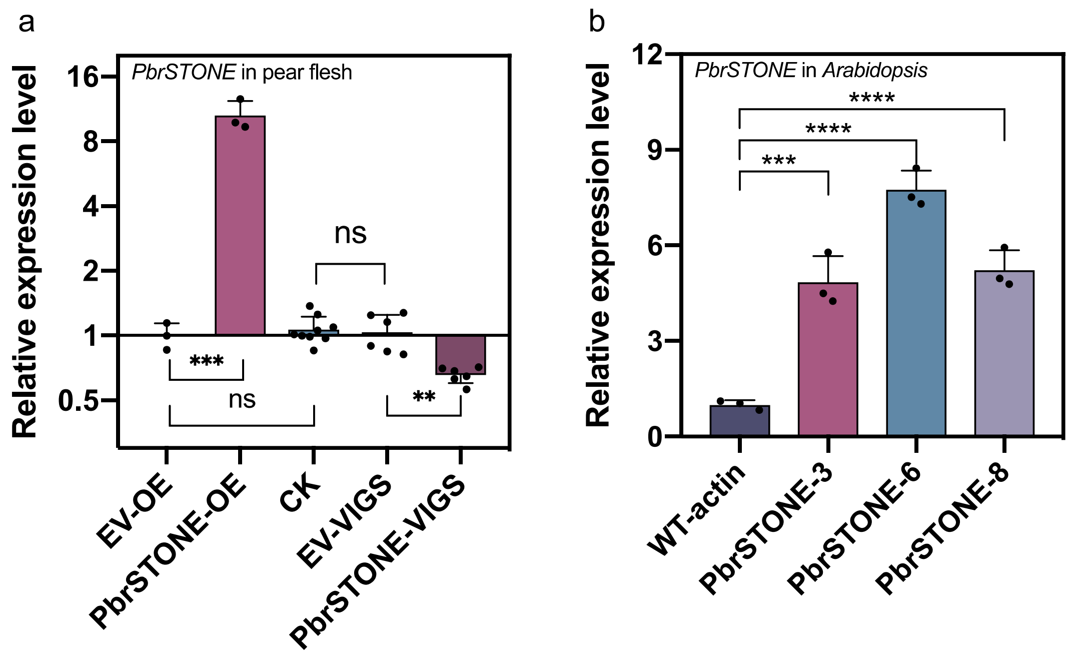

Supplementary Figure 18. Expression levels of *PbrSTONE* in transgenic pear and *Arabidopsis*. **a**. Relative expression levels of *PbrSTONE* in the flesh tissue around the infiltration sites with empty vector (*EV-OE* and *EV-VIGS*), *PbrSTONE* overexpression construct (*PbrSTONE-OE*), *PbrSTONE* silencing construct (*PbrSTONE-VIGS*) and non-infiltrated site (CK). n=3, 3, 9, 6 and 6 biologically independent samples for *EV-OE*, *PbrSTONE-OE*, CK, *EV-VIGS* and *PbrSTONE-VIGS*, respectively. **b**. *PbrSTONE* expression levels in wild type and transgenic *Arabidopsis* lines overexpressing *PbrSTONE*. n=3 biologically independent samples. Data presented are mean ± SD. *p*-values were determined by two-tailed Student's t-test (**$p < 0.01$, ***$p < 0.001$, ****$p < 0.0001$; ns, not significant). Source data are provided as a Source Data file.

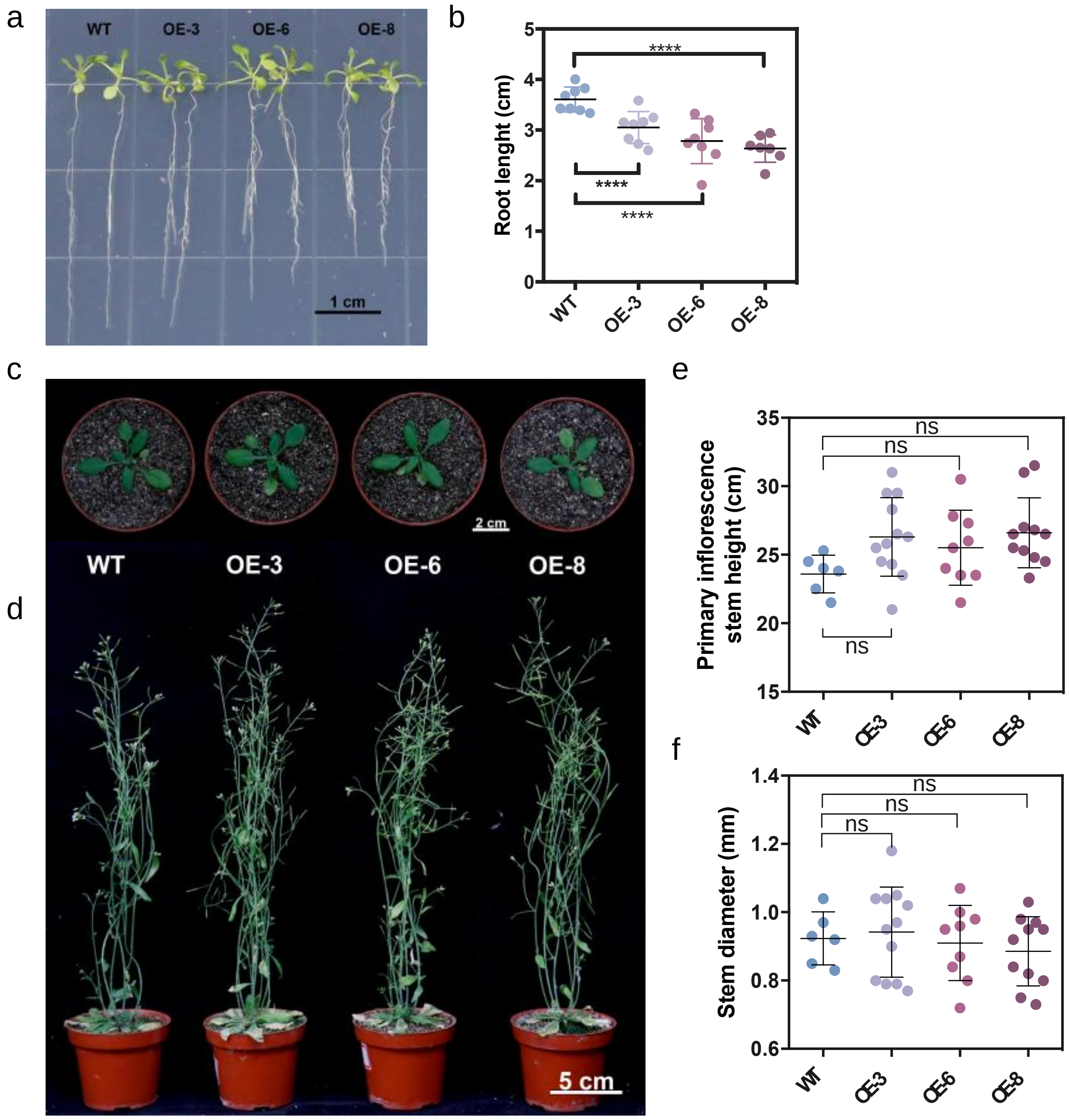

Supplementary Figure 19. Phenotype of the transgenic *Arabidopsis* overexpressing *PbrSTONE*. **a-b**. Root length of WT and *PbrSTONE*-overexpression *Arabidopsis* plants. n=8, 8, 8 and 7 biologically independent samples for WT, OE-3, OE-6 and OE-8, respectively. **c-f**. Primary inflorescence stem height (**c** and **e**) and stem diameter (**d** and **f**) in *PbrSTONE*-overexpression *Arabidopsis* lines and wild type (WT) plants. n=6, 12, 9 and 11 biologically independent samples for WT, OE-3, OE-6 and OE-8, respectively. Data presented are mean ± SD. *p*-values were determined by two-tailed Student's t-test (*****p*< 0.0001; ns, not significant). Source data are provided as a Source Data file.

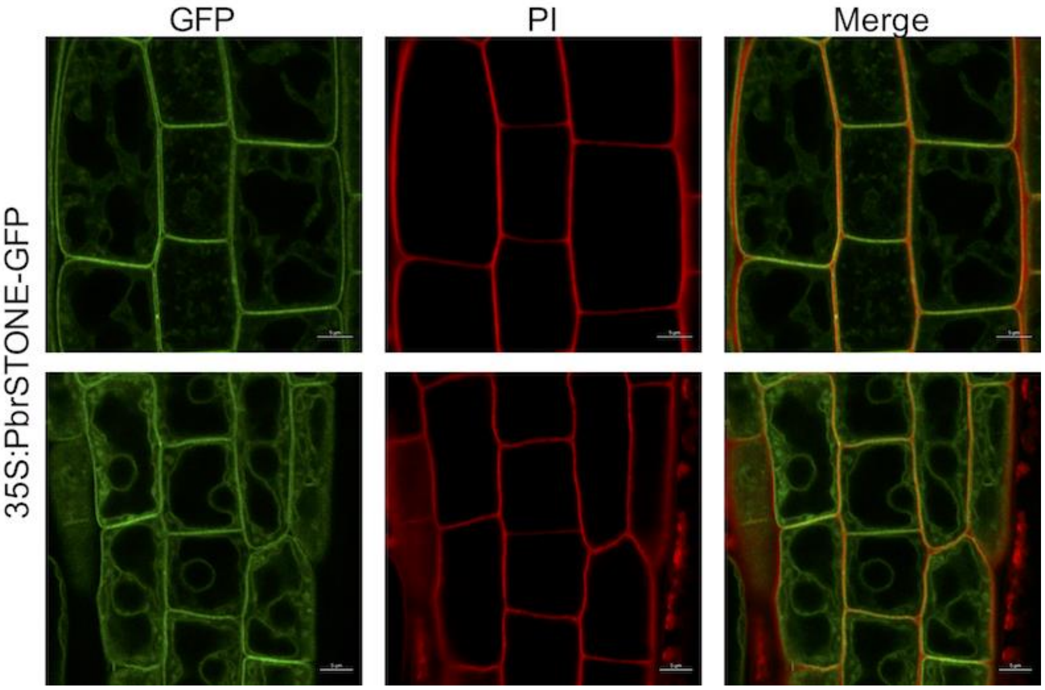

Supplementary Figure 20. Subcellular localization of PbrSTONE in transgenic Arabidopsis root. The localization of PbrSTONE protein in cytoplasm and cytomembrane was determined using PbrSTONE-GFP fusion protein. The cell wall was identified with Propidium iodide (PI) staining. The experiment was repeated two times independently with similar results obtained.

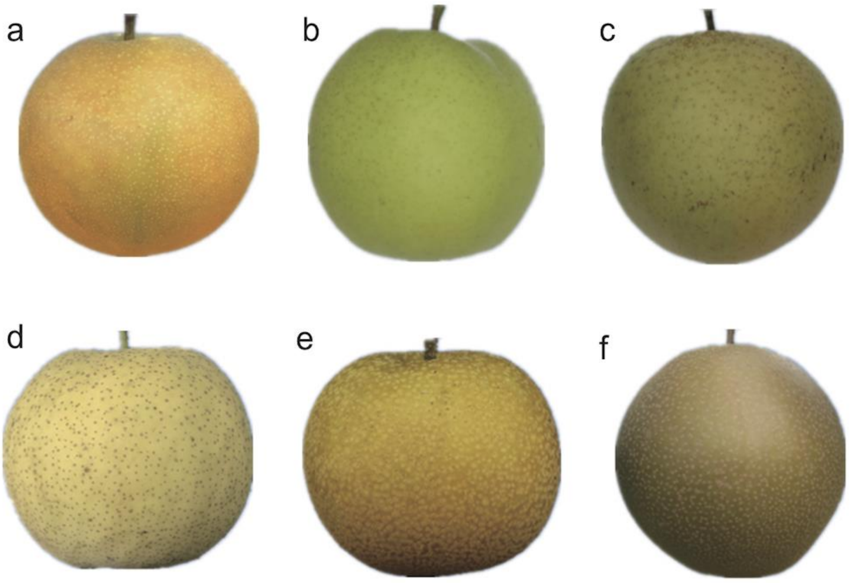

Supplementary Figure 21. Diversity of fruit skin color. **a**. ‘1’, Yellow; **b**. ‘2’, Green; **c**. ‘3’, Yellow-green; **d**. ‘4’, Green-yellow; **e**. ‘5’, Yellow-brown; **f**. ‘6’, Brown.

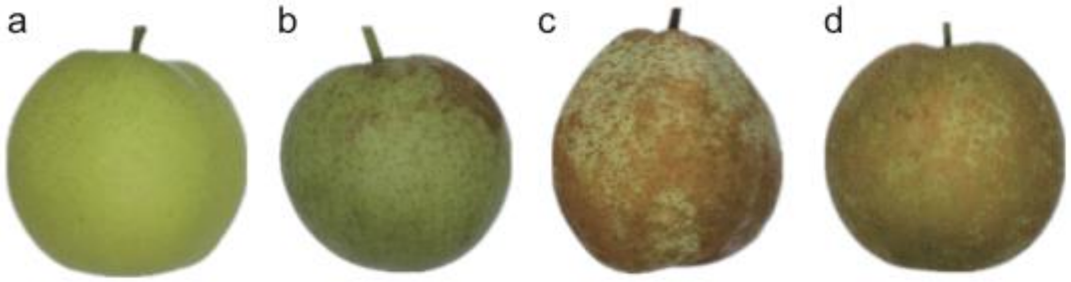

Supplementary Figure 22. Different coverage ratio of fruit russet. **a**. ‘1’, ratio<1/16; **b**. ‘3’: 1/16≤ratio<1/8; **c**. ‘5’: 1/8≤ratio<1/4; **d**. ‘7’: ra3tio≥1/4.

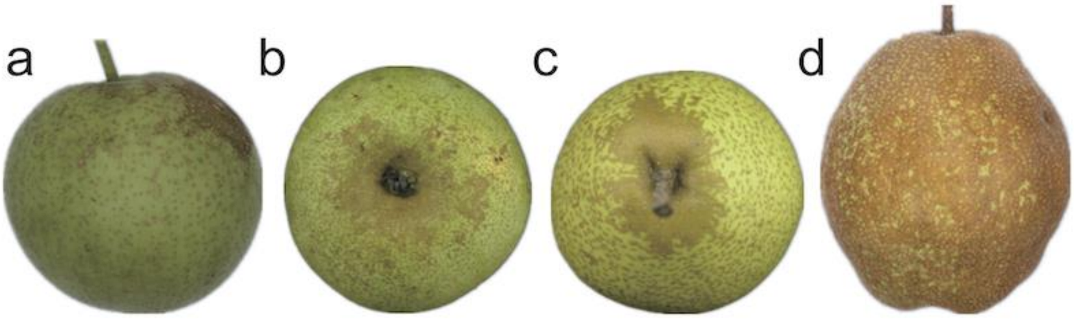

Supplementary Figure 23. Different location of fruit russet. **a**. ‘1’, Sunny side; **b**. ‘2’, Sepal side; **c**. ‘3’, Stem side; **d**. ‘4’, Whole fruit.

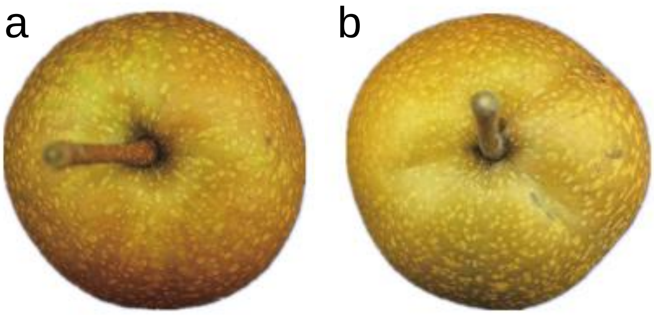

Supplementary Figure 24. Furrows on fruit surface. **a**. ‘0’, none; **b**. ‘1’, existence.

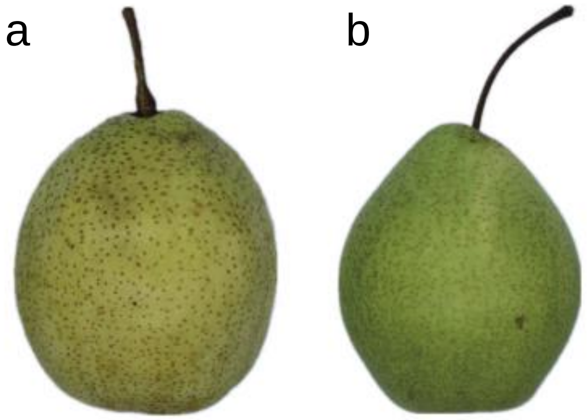

Supplementary Figure 25. Different direction of pear carpopodium. **a**. ‘1’, vertical carpopodium; **b**. ‘2’, oblique carpopodium

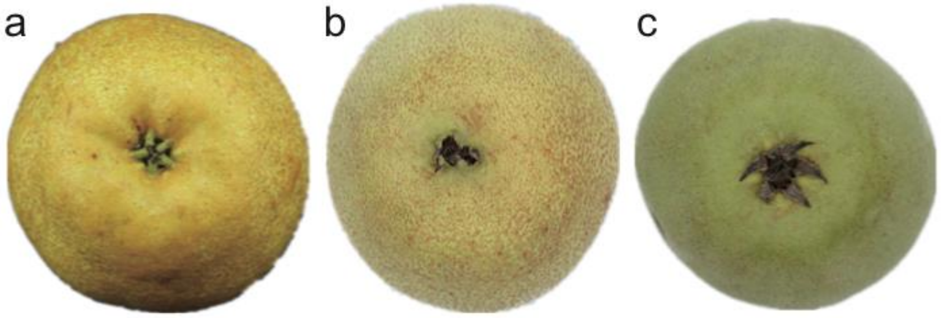

Supplementary Figure 26. Different direction of pear sepal. **a**. '1', Sepal get-together; **b**. '2', Vertical sepal; **c**. '3', Radiational sepal.
