# Supplementary material for: Genome-wide association studies provide insights into the genetic determination of fruit traits of pear
Source: Nat Commun. 2021 Feb 18;12:1144. doi: 10.1038/s41467-021-21378-y (PMC7892570; doi:10.1038/s41467-021-21378-y)
Supplement: Supplementary file 2 — Reporting Summary [file 41467_2021_21378_MOESM2_ESM.pdf]

Corresponding author(s): Jun Wu, Shaoling Zhang, Zhangjun Fei

Last updated by author(s): Jan 21, 2021

Reporting Summary

Nature Research wishes to improve the reproducibility of the work that we publish. This form provides structure for consistency and transparency in reporting. For further information on Nature Research policies, see our Editorial Policies and the Editorial Policy Checklist.

Statistics

For all statistical analyses, confirm that the following items are present in the figure legend, table legend, main text, or Methods section.

n/a	Confirmed	
☐	☒	The exact sample size (*n*) for each experimental group/condition, given as a discrete number and unit of measurement
☐	☒	A statement on whether measurements were taken from distinct samples or whether the same sample was measured repeatedly
☐	☒	The statistical test(s) used AND whether they are one- or two-sided<br>*Only common tests should be described solely by name; describe more complex techniques in the Methods section.*
☒	☐	A description of all covariates tested
☐	☒	A description of any assumptions or corrections, such as tests of normality and adjustment for multiple comparisons
☐	☒	A full description of the statistical parameters including central tendency (e.g. means) or other basic estimates (e.g. regression coefficient) AND variation (e.g. standard deviation) or associated estimates of uncertainty (e.g. confidence intervals)
☐	☒	For null hypothesis testing, the test statistic (e.g. *F*, *t*, *r*) with confidence intervals, effect sizes, degrees of freedom and *P* value noted<br>*Give P values as exact values whenever suitable.*
☒	☐	For Bayesian analysis, information on the choice of priors and Markov chain Monte Carlo settings
☒	☐	For hierarchical and complex designs, identification of the appropriate level for tests and full reporting of outcomes
☒	☐	Estimates of effect sizes (e.g. Cohen's *d*, Pearson's *r*), indicating how they were calculated

Our web collection on statistics for biologists contains articles on many of the points above.

Software and code

Policy information about availability of computer code

Data collection	No software was used in data collection.
Data analysis	We only used freely available bioinformatics software for our data analysis.<br>Trimmomatic (0.39), BWA (0.7.17-r1188), Genome Analysis Toolkit (4.1.4), vcftools (0.1.15), SweeD (3.3.1), STRUCTURE (2.3.4), TASSEL (5), EMMAX, CLUSTAL (2.1), IQ-TREE55 (1.6.10)

For manuscripts utilizing custom algorithms or software that are central to the research but not yet described in published literature, software must be made available to editors and reviewers. We strongly encourage code deposition in a community repository (e.g. GitHub). See the Nature Research guidelines for submitting code & software for further information.

Data

Policy information about availability of data

All manuscripts must include a data availability statement. This statement should provide the following information, where applicable:

- Accession codes, unique identifiers, or web links for publicly available datasets
- A list of figures that have associated raw data
- A description of any restrictions on data availability

Raw genome resequencing reads have been deposited into the NCBI sequence read archive (SRA) under project of PRJNA563813. Sequence data in this article can be found at GenBank with the following accession numbers: MT711883 (PbrSTONE) and MT711884 (PbrC3H1). SNPs identified in this study are available at https://zenodo.org/deposit/3971245 (DOI: 10.5281/zenodo.3971245). The source data underlying Figs 4c, d, 5a-z and Supplementary Figs S4, S17, S18a, b and S19 are provided as a Source Data file.

Field-specific reporting

Please select the one below that is the best fit for your research. If you are not sure, read the appropriate sections before making your selection.

- [x] Life sciences
- [ ] Behavioural & social sciences
- [ ] Ecological, evolutionary & environmental sciences

For a reference copy of the document with all sections, see nature.com/documents/nr-reporting-summary-flat.pdf

Life sciences study design

All studies must disclose on these points even when the disclosure is negative.

Sample size	A total of 312 pear accessions were used in this study, which are sufficient for the GWAS analysis
Data exclusions	No data were excluded.
Replication	The pear phenotype data were collected in 2014, 2015, and 2016 for three years (some traits were recorded in two years because of the unpredictable dropped fruits). At least three independent lines over-expressing PbrSTONE in pears and Arabidopsis were used for the analysis, and the consistent results were presented in the manuscript.
Randomization	Validation of identified SNPs were performed by randomly selected a subset of SNPs.
Blinding	For phenotype data collection, the investigators were not blinded to the pear accessions' name. For the genotype data generating, the investigators were blinded to the accessions' name.

Reporting for specific materials, systems and methods

We require information from authors about some types of materials, experimental systems and methods used in many studies. Here, indicate whether each material, system or method listed is relevant to your study. If you are not sure if a list item applies to your research, read the appropriate section before selecting a response.

Materials & experimental systems

n/a	Involved in the study	
x		Antibodies
x		Eukaryotic cell lines
x		Palaeontology and archaeology
x		Animals and other organisms
x		Human research participants
x		Clinical data
x		Dual use research of concern

Methods

n/a	Involved in the study	
x		ChIP-seq
x		Flow cytometry
x		MRI-based neuroimaging
