# Supplementary material for: Genome-wide association studies provide insights into the genetic determination of fruit traits of pear
Source: Nat Commun. 2021 Feb 18;12:1144. doi: 10.1038/s41467-021-21378-y (PMC7892570; doi:10.1038/s41467-021-21378-y)
Supplement: Supplementary file 3 — Description of Additional Supplementary Files [file 41467_2021_21378_MOESM3_ESM.pdf]

Description of Additional Supplementary Files

Supplementary Data 1. Summary statistics of sequencing and mapping for 312 pear accessions
Supplementary Data 2. NGS and Sanger sequencing to validate SNP calling
Supplementary Data 3. Candidate selective sweep regions
Supplementary Data 4. Genes in the selective sweep regions
Supplementary Data 5. Phenotypic data of eleven fruit traits in sand pears
Supplementary Data 6. Main candidate regions for pear fruit traits based on GWAS
Supplementary Data 7. GWAS predicted candidate genes
Supplementary Data 8. Primers used in gene function validation
